# Pan-Cancer DNA Methylation Analysis and Tumor Origin Identification of Carcinoma of Unknown Primary Site Based on Multi-Omics

**DOI:** 10.3389/fgene.2021.798748

**Published:** 2022-01-06

**Authors:** Pengfei Liu

**Affiliations:** ^1^ State Key Laboratory of Genetic Engineering, Collaborative Innovation Center For Genetics and Development, School of Life Sciences, Fudan University, Shanghai, China; ^2^ Department of Biostatistics and Computational Biology, School of Life Sciences, Fudan University, Shanghai, China

**Keywords:** multi-omics, DNA methylation, cancer of unknown primary, metastasis, tissue-specific classifier

## Abstract

The metastatic cancer of unknown primary (CUP) sites remains a leading cause of cancer death with few therapeutic options. The aberrant DNA methylation (DNAm) is the most important risk factor for cancer, which has certain tissue specificity. However, how DNAm alterations in tumors differ among the regulatory network of multi-omics remains largely unexplored. Therefore, there is room for improvement in our accuracy in the prediction of tumor origin sites and a need for better understanding of the underlying mechanisms. In our study, an integrative analysis based on multi-omics data and molecular regulatory network uncovered genome-wide methylation mechanism and identified 23 epi-driver genes. Apart from the promoter region, we also found that the aberrant methylation within the gene body or intergenic region was significantly associated with gene expression. Significant enrichment analysis of the epi-driver genes indicated that these genes were highly related to cellular mechanisms of tumorigenesis, including T-cell differentiation, cell proliferation, and signal transduction. Based on the ensemble algorithm, six CpG sites located in five epi-driver genes were selected to construct a tissue-specific classifier with a better accuracy (>95%) using TCGA datasets. In the independent datasets and the metastatic cancer datasets from GEO, the accuracy of distinguishing tumor subtypes or original sites was more than 90%, showing better robustness and stability. In summary, the integration analysis of large-scale omics data revealed complex regulation of DNAm across various cancer types and identified the epi-driver genes participating in tumorigenesis. Based on the aberrant methylation status located in epi-driver genes, a classifier that provided the highest accuracy in tracing back to the primary sites of metastatic cancer was established. Our study provides a comprehensive and multi-omics view of DNAm-associated changes across cancer types and has potential for clinical application.

## Introduction

Although most cancer patients are defined as having primary tumors at the early stage of cancer diagnosis, 10%–15% of cancer patients are still diagnosed with cancer cell metastasis (Greco 2013). Among them, about one-third of patients may be diagnosed with the primary site of the tumor (Sokilde et al., 2014). Therefore, metastatic cancer with CUP accounts for 30%–60% of all cancer diagnoses, and is considered the seventh most common cancer subtype (Sokilde et al., 2014). Since effective cancer treatment relies on the early identification of the tumor original sites, patients with CUP have a poor prognosis, a median survival time of 9 months (95% CI: 8.3–10.0), and a 1-year survival rate of less than 25% (Massard et al., 2011). In clinical medicine, the cancers of unknown primary site are a molecularly heterogeneous cancer, which makes interpretation of histomorphology and immunohistochemistry difficult (Moran et al., 2016). Therefore, we need to screen a set of cancer tissue-specific biomarkers to identify the primary site of metastatic cancer and improve the treatment and prognosis of patients with CUP.

Epi-driver gene refers to the abnormal changes of gene expression or cell phenotype caused by mechanisms other than changes in the DNA sequence, which are changed through changes in DNAm or chromatin modification, and persist with the division of tumor cells (Vogelstein et al., 2013). DNAm is one of the most common epigenetic events. In the past few years, abnormal DNA methylation has been shown to play a key role in a variety of diseases (Liu & Shi 2017; Sui et al., 2021; Wang et al., 2017; Wu et al., 2017). In terms of tumors, abnormal methylation can be used as a biomarker for clinical decision-making, diagnosis, and prognosis of different cancers (Rajaraman et al., 2015; Wei et al., 2015; Hao et al., 2017; Kirby et al., 2017). However, research on DNA methylation mainly focuses on the promoter region and CpG islands. The anomalous changes of DNA methylation located in non-CpG islands or gene body region may also play an important role in gene expression regulation (Jjingo et al., 2012; Rao et al., 2013). In addition, tissue-specific gene expression is also related to differences in DNA methylation along the shelf/shore of CpG islands in different tissues (Irizarry et al., 2009b). Although some studies have investigated the DNA methylation profile of human pan-carcinoma (Irizarry et al., 2009b; Yang et al., 2017), there are still no biomarkers for the diagnosis of cancer subtype or the determination of the primary sites of metastatic cancer. On the other hand, cancer cells undergo significant metabolic changes to adapt to the growth characteristics of cancer cells or the tissue environment, such as the “Warburg” effect. This metabolic reprogramming promotes the invasion and metastasis of cancer cells, and causes the immunosuppression of cancer cells. One of the most important reasons leading to the metabolic reprogramming is the abnormal expression of a large number of enzyme-coding genes involved in a variety of metabolic pathways (Chen et al., 2013). However, it is precisely because of this significant metabolic reprogramming that gene expression analysis based on known pathways is insufficient to detect abnormal gene expression patterns. Therefore, we would integrate gene expression and biological networks (including metabolic networks and protein–protein interaction networks), comprehensively explore the DNA methylation pattern of pan-cancer tissues, deeply understand the methylation mechanism of cancer cells, and screen potential molecular markers for the identification of cancer subtypes and the tumor original sites of metastatic cancer.

In this study, we first explored and screened the epi-driver CpG sites that had a strong correlation between DNA methylation and gene expression based on the genome-wide DNA methylation profile and mRNA expression profile. These epi-driver CpG sites in cancer cells lead to abnormal gene expression, making them unable to control the cell cycle, cell apoptosis, and/or DNA repair. Based on multi-omics strategies, we further integrate gene co-expression network (Langfelder & Horvath 2008), enzyme metabolism (https://www.gemome.jp) and protein–protein interaction network (https://string-db.org) to screen the epi-driver genes that cause metabolic reprogramming, and abnormal gene expression. Based on XGBoost and SHAP algorithms, the six epi-driver CpG sites located in five genes were identified as the best biomarkers. The random forest model was constructed to identify cancer subtypes and trace the tumor original site with high tissue specificity (AUC > 95%). This will provide clinical evidence for the identification of primary tumor subtypes and cancer tissue-specific treatment, and contribute to improving patient survival.

## Materials and Methods

### Workflow Chart and Samples Preparation

**FIGURE 1:**
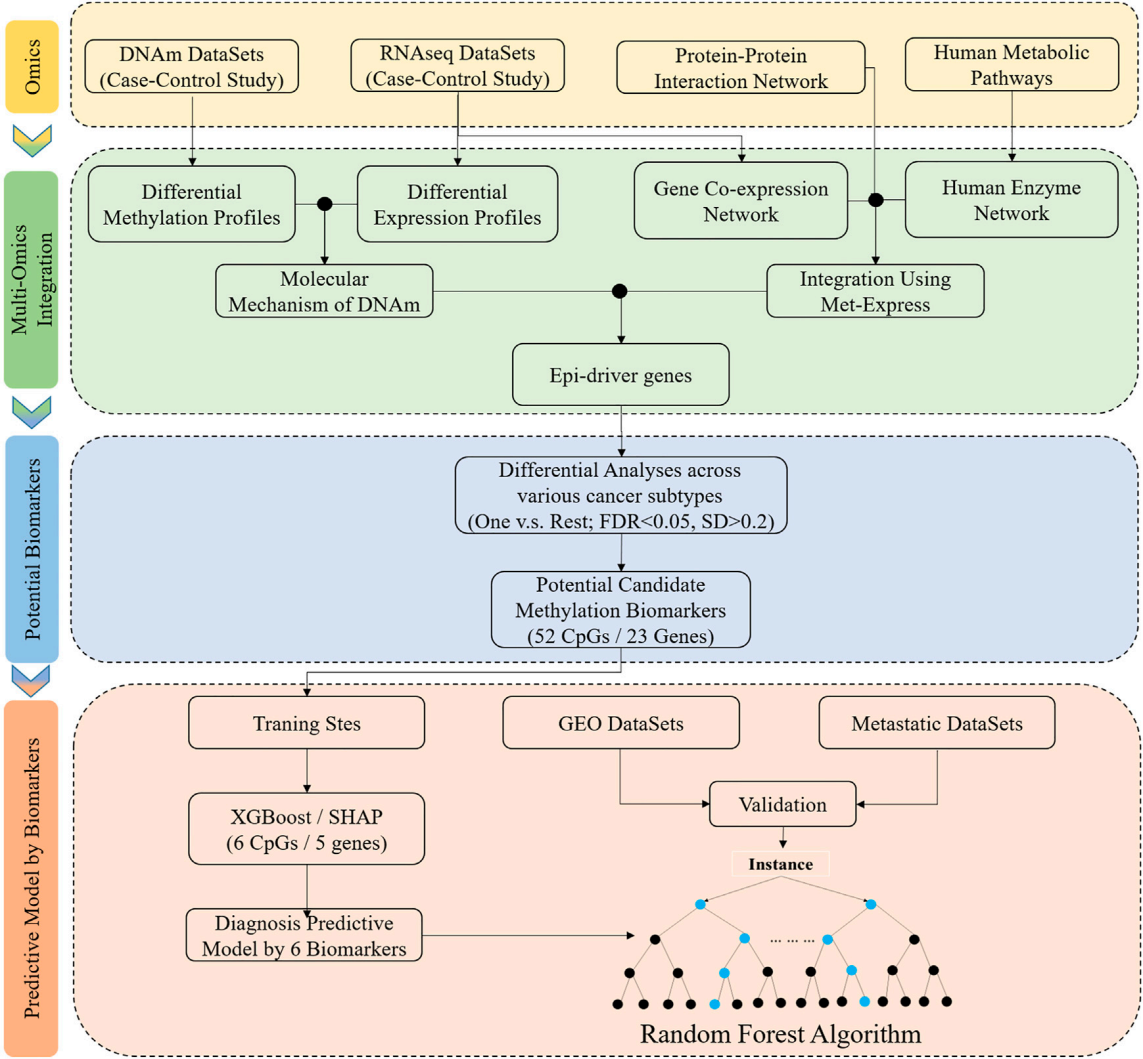
Schematic overview of the workflow of data analysis and the development of classifier based on tissue-specific DNA methylation markers. DNAm, DNA methylation; One vs. Rest, a given tumor tissue and other tumor tissue types; FDR and SD, the differential analyses were performed between a given tumor tissue and other tumor tissue types, and the significant difference was defined: the FDR (Bonferroni-adjusted *p*-value) was less than 0.05 and standard deviation (SD) was greater than 0.2. Integrated methylation signatures on tumor tissues and matched adjacent normal tumor tissues was used to identify the epi-driver genes. Potential biomarkers: One vs. Rest method was applied to identify the potential candidate methylation biomarkers. Predictive model by biomarkers: XGBoost and SHAP algorithms was used to a training cohort to identify a final selection of six biomarkers. These six biomarkers were used to construct the classifier, and the independent datasets (GEO datasets and metastatic datasets) were used to evaluate performance.

Figure 1 shows the study workflow chart, including the algorithm flow. Those tumor tissues and adjacent normal tissues were acquired from The Cancer Genome Atlas pilot (TCGA) project (https://tcga-data.nci.nih.gov/tcga/) for this study, which were divided into two subgroups. One subgroup of these datasets was used as the training sets to filter features and construct classifier, and the testing sets used another subgroup of these datasets to assess the performance of the classifier.

In order to screen the effective biomarkers, the strict review was performed to only filter those datasets that met the specific rules for our research. The criteria included that the datasets have both DNAm and mRNA profiles, a sufficient number of samples for tumor tissue and matched adjacent normal tissue (≥10 pairs of samples), and completely similar clinical information (Table 1; Supplementary Table S1). After filtering, 5,600 samples for DNAm-based profiles, namely, 4,956 tumor samples and 644 normal samples, and 6,351 samples for mRNA-based profiles, namely, 5,776 tumor samples and 575 normal samples, were selected from TCGA project. Of which, 342 pairs of samples have both DNAm-based profiles and mRNA-based profiles with both tumor and matched adjacent normal tissues. DNAm profiles based on the Illumina Infinium HumanMethylation450 (HM450) BeadChips were obtained, which were involved in more than 480,000 CpG sites in the human genome. Gene expression profiles (RNA-seq datasets) were generated using the HTSeq pipeline from TCGA and were quantified using HTSseq-Counts.

**TABLE 1:** Summary of TCGA cancer type and number of samples used in each analysis.

Cancer type	TCGA project	RNA-seq				Illumina human methylation 450				RNA-seq vs. Methylation	
		Unpaired		Paired		Unpaired		Paired		Paired^a^	
		Normal	Tumor	Normal	Tumor	Normal	Tumor	Normal	Tumor	Normal	Tumor
Bladder urothelial carcinoma	BLCA	0	393	19	19	0	396	21	21	17	17
Breast-invasive carcinoma	BRCA	1	989	112	112	6	702	90	90	76	76
Colon adenocarcinoma	COAD	0	434	41	41	0	275	38	38	19	19
Head and neck squamous cell carcinoma	HNSC	1	459	43	43	0	480	50	50	20	20
Kidney clear cell renal cell carcinoma	KIRC	0	467	72	72	0	165	160	160	24	24
Kidney renal papillary cell carcinoma	KIRP	1	258	31	31	0	231	45	45	22	22
Liver hepatocellular carcinoma	LIHC	0	324	50	50	0	330	50	50	41	41
Lung adenocarcinoma	LUAD	2	468	57	57	3	437	29	29	18	18
Prostate adenocarcinoma	PRAD	0	445	52	52	0	453	50	50	35	35
Thyroid carcinoma	THCA	0	452	58	58	0	459	56	56	49	49
Uterine corpus endometrial carcinoma	UCEC	12	529	23	23	13	406	33	33	21	21
Total		17	5218	558	558	22	4334	622	622	342	342

a NoteThe patients have both gene expression profiles and methylation profiles.

In addition, the human KEGG Markup Language (KGML) data were generated by KEGG project (http://www.genome.jp/kegg/) to filter the key enzyme-coding genes. The protein–protein interaction network was collected from the human protein reference database (STRING database, http://www.hprd.org/) to select the hub genes.

### Differential Analysis Based on DNA Methylation and Gene Expression Profiles

For DNA methylation profiles, the Mann–Whitney *U* test (called Wilcoxon Rank Sum Test) was used to select the significant differentially methylated CpG sites between tumor tissues and matched adjacent normal tissues (Selamat et al., 2012). Prior to analysis, data preprocessing was executed to remove the invalid or ambiguous CpG sites. The CpG sites located in chromosomes X and Y, identified as non-unique in the genome and known as single-nucleotide polymorphisms, were eliminated (Cai et al., 2020; Noushmehr et al., 2010; Selamat et al., 2012). The CpG sites with more than 50% missing values were excluded, and the remaining missing values were imputed based on K-Nearest Neighbor algorithm (Wu et al., 2020). The CpG sites were identified as statistically significantly different between tumor tissues and matched adjacent normal tissues with adjusted *p*-value < 0.05, and the absolute value of the mean *β* value difference between groups was greater than 0.2 (adjusted *p*-value < 0.05 and |△*β*| > 0.2) (Bjerre et al., 2020).

For gene expression profiles, the differential analysis was performed between tumor tissues and matched adjacent normal tissues. The read counts of genes were used as an input expression matrix to “limma” package in Bioconductor 3.10. Genes were identified as statistically significantly different between tumor tissues and matched adjacent normal tissues with adjusted *p*-value < 0.05, and the absolute value of mean log2 fold-change was greater than 2 (adjusted *p*-value < 0.05 and |log2FC| > 2).

### DNA Methylation Patterns Through Integrating Gene Expression

For each of a number of specified genomic region windows in relation to genes, we constructed a DNA methylation (*β*-values of CpG sites) matrix by annotating for every CpG site within the given gene region (including 0–1,500 up-/downstream of TSS sites and gene body region in the GRCh38). When a CpG existed in a given gene region, we defined that the CpG was associated with the gene. For the set of CpG sites associated with a given gene within a specified region in proximity to the gene (e.g., 0–1500 bp upstream of TSS sites, 0–1500 bp downstream of TSS sites or within the gene body), correlation between DNA methylation status and gene expression profiles for each CpG sites or genes was calculated using linear regression model in all the samples. In addition to modeling expression as a function of methylation event, models incorporating cancer type as a factor in addition to methylation event were also considered. Genes for which methylation event were significant (adjusted *p*-value <0.05) after correcting for cancer type were explored.

### Identification of Epi-Driver Genes Based on Multi-Omics

Epi-driver genes have two important characters: aberrant expression of gene in the cancer cells and selective advantage in terms of the growth of tumor cells (Vogelstein et al., 2013). The former can be easily obtained by analyzing the difference of transcriptome, while the latter is more complex, and there is no direct method to calculate and evaluate. In our previous research, we developed a bioinformatics method based on the integration of multi-omics data, and namely Met-express (Chen et al., 2013). In this method, a cancer gene co-expression network was constructed by using transcriptome data of tumor tissue and matched adjacent normal tissue, and then a tumor-specific co-expression module was obtained through module division. Then, based on the relationship between products and substrates of enzyme in the metabolic reactions, the directed network of enzyme genes was constructed, which connected the enzyme genes of upstream metabolic reactions to the enzyme genes of downstream metabolic reactions directionally, and calculated the connection degree between each enzyme gene and downstream enzyme genes. Through computing an importance score (Score_A_) for a given enzyme-coding gene (Chen et al., 2013), Met-express can obtain upstream enzyme genes that had significant co-expression pattern with downstream enzyme genes in the tumor-specific co-expression module, and these genes were identified as the key enzyme genes with significant abnormal expression in the metabolic reprogramming. It has been proven that the abnormal expression of key enzyme genes played an important role in the abnormal growth of tumor cells (Chen et al., 2013). Therefore, the method developed by us provided a new way to search for tumor driver genes. Because of the universality of integration method, we replaced enzyme metabolism network with protein–protein interaction network (STRING, http://www.hprd.org/) to predict the new driver genes/proteins based on the protein–protein interaction network.

$$Score_{A}=\left|AUC_{ROC}-0.5\right|\times \log_{2}\left(\frac{C_{in}/C_{all}}{N_{in}/N_{all}}\right)$$

Here, Score_A_ refers to the importance score of a given enzyme-coding gene A. C_in_ and C_all_ refer to the number of within-module genes and the number of all genes that are connected from A in the metabolic network or protein–protein interaction network, respectively. N_in_ and N_all_ refer to the number of within-module genes, and the number of all genes in the co-expression network, respectively.

In the discovery, we would overlap the driver genes predicted by two biological networks with the abnormally expressed genes from difference analysis to obtain all possible cancer driver genes. Based on linear regression model after correcting for cancer type, the correlation analysis between methylation status and gene expression level in the same gene was performed to select the significantly methylated genes with the abnormal gene expression. Finally, we performed overlap analysis between those driver genes, and the significantly methylated genes with the abnormal gene expression to identify tissue-specific epi-driver genes with abnormal expression.

### Screening of Candidate Tissue-specific Diagnostic Biomarkers

For DNAm-based profiles, the primary feature selection was conducted by Mann–Whitney *U* test and Met-express algorithm. Some significant differentially methylated CpG sites located within epi-driver genes were identified to reduce redundant methylation sites of original datasets. Subsequently, we conducted a second feature screening to select tissue-specific CpG sites. The significantly methylated CpG sites between the given tumor tissue and other tumor tissue types [one versus all, Student’s *t*-test] were obtained, and the significant difference was defined: the FDR-value was less than 0.05 and the standard deviation (SD) was greater than 0.2 (Ding et al., 2019). Finally, the tissue-specific CpG sites located within epi-driver genes were selected, which played an important role in tumor progression, such as causing metabolic reprogramming.

DNA methylation profiles included more than 1,600 CpG sites after the preprocessing procedure. Consequently, those CpG sites selected by second-level feature selection were still large in number and redundant. Therefore, we further performed feature selection to identify tissue-specific CpG sites by XGBoost and SHAP algorithm. Apart from that, the reason why the second-level feature selection is used is to make the model easier to explain, delete redundant variables without improving performance, and reduce the complexity of the model to avoid over-fitting.

### Classifier Construction and Performance Evaluation

The best CpG sites obtained from the secondary feature selection were regarded as biomarkers specific to each type of tissue. In order to construct the tissue-specific random forest model, all classes were integrated. Because selecting the related biomarkers (such as SNPs, gene expression profiles, and methylation profiles) for sample classification (such as distinguishing cancer patients from non-cancer patients) is a common goal of most omics studies, another main goal of our research is to identify a smaller set of biomarkers that could be used for clinical diagnosis. Therefore, we need to minimize the number of biomarkers to exclude “redundant” biomarkers, and at the same time, we need to have higher prediction performance (Díaz-Uriarte & Alvarez de Andrés 2006). In view of the uniqueness of this study and the dimension of epigenome data, those classification algorithms, which are used for two-class and multi-class tasks, or when the number of features is more than the observations, and which avoid over-fitting, would be very interesting. Random forest has been proven to have a better performance in many classification cases (Breiman 2001; Statnikov et al., 2008). Therefore, the random forest algorithm was used to construct a multi-class classifier for identifying cancer subtypes.

We divided the cancer datasets into a training set and a testing set according to a ratio of 7:3, which were used to build a classifier model and evaluate performance. In addition, because the number of samples of one subtype (given tissue class) was smaller compared with samples of other subtypes (other tissue classes), it would lead to imbalance. In order to solve this problem, which seriously affects the performance of the classifier, the under-sampling (Al-Shahib et al., 2005) algorithm was used, which randomly selected a subset from the multi-class samples to form a balanced dataset with the corresponding single tissue class. Based on the balanced datasets, a multi-class classifier was trained to distinguish cancer subtypes (one versus all). Tenfold cross-validation was used in the training process of multi-class classifier, and the performance of the multi-class classifier was evaluated based on the area under the ROC curve (AUC).

### Classifier Validation Based on Metastatic Cancer and Independent GEO Datasets

For the further validation of the performance of the classifier, the Illumina HumanMethylation450 BeadChip data were comprehensively queried from GEO (Gene Expression Omnibus, https://www.ncbi.nlm.nih.gov/geo/). In the process of data retrieval, the following conditions should be met as much as possible which the patients without receive neoadjuvant therapy. GSE69914 (Breast Invasive carcinoma, 305 tumor tissues without metastases), GSE48684 (Colon Adenocarcinoma, 88 tumor tissues without metastases), GSE89582 (Liver Hepatocellular Carcinoma, 37 tumor tissues without metastases), GSE66836 (Lung Adenocarcinoma, 164 tumor tissues without metastases), and GSE73549 (Pancreatic Adenocarcinoma, 57 tumor tissues without metastases) were for the tissue-specific classifier. The validation datasets for tumor with metastases were obtained, which were GSE58999 (43 breast cancers with lymph node metastases) and GSE73549 and GSE38240 (26 prostate cancers with lymph node metastases), to evaluate the performance of identifying primary tumor sites.

### Functional Enrichment Analyses

To investigate the functions of CpG sites or genes, Gene Ontology (GO) enrichment analysis was performed using “clusterProfiler” package (R version 4.0.2), including identification of terms in the biological process (BP). In enrichment analysis, all the detected genes were used as background gene sets, and the selected genes of interest were used as query gene sets. The GO-terms with BH-adjusted *p*-value < 0.05 were considered as statistical significance. The online database *GeneMANIA* (http://genemania.org, version 3.6) was used to explore biological function at the protein level with an interaction score >0.4 as the cutoff value. Afterward, the network was visualized in software Cytoscape (version 3.6.1).

### Statistical Analysis

The Mann–Whitney *U* test was used to identify the significantly methylated CpG sites between tumor tissues and matched adjacent normal tissues (adjusted *p*-value < 0.05 and |△*β*| > 0.2), and the Bayesian algorithm based on “limma” package was used to identify significantly expressed genes between tumor tissues and matched adjacent normal tissues (adjusted *p*-value < 0.05 and |log2FC| > 2). For all cancers, the linear regression models corrected by cancer subtypes were used to associate the gene expression with nearby CpG sites. Genes for CpG sites were considered as significant when the adjusted *p*-value was less than 0.05. The permutation testing was used to verify the non-random associations between methylation events and gene expression. For the numbers of overlapping epi-driver genes between any two cancer subtypes, the significance of overlap was calculated using one-sided Fisher’s extract test. All *p*-values were adjusted using the Benjamini/Hochberg method (BH), and the significance was defined when the *p*-value after adjustment is less than 0.05. The classifier was constructed in scikit-learn framework (version 0.20.3) with the “sklearn.ensemble.RandomForestClassifier” algorithm. The hyper-parameters in random forest algorithm were optimized based on Out of Bag (OOB) score, which were the number of trees (10–100) and the criterion (Gini coefficient and Entropy). The unsupervised hierarchical clustering was carried out using Euclidean distance matrix and complete-linkage method. The default values were used when other parameters were not provided in functions. All statistical analyses were performed with R version 4.0.2 and Python 3.8.

## Results

### DNA Methylation Patterns Associated With Cancer Progression Across Cancer Types

**FIGURE 2:**
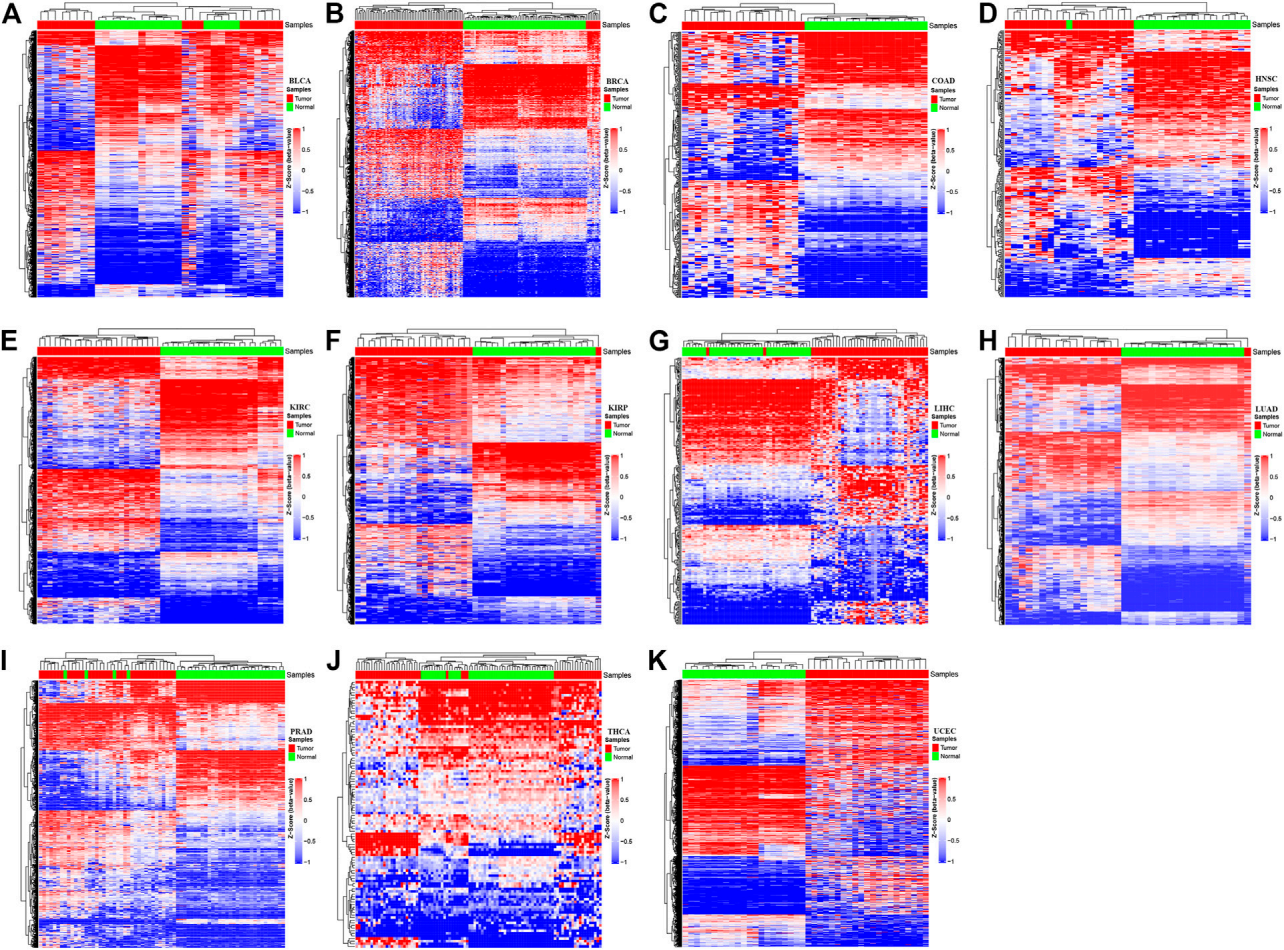
The methylation profiles in tumor tissues versus the adjacent-match normal tissues for each of the 11 cancer types in The Cancer Genome Atlas (TCGA). The Hierachical Clustering was executed by means of Euclidean distance based on the methylation status of various cancer types. *Z*-score was calculated based on the methylation levels of each cancer type. The red color represents hyper-methylation and the blue color represents hypo-methylation. The letters represent the different cancer types: **(A)** Bladder Urothelial Carcinoma; **(B)** Breast invasive carcinoma; **(C)** Colon adenocarcinoma; **(D)** Head and Neck squamous cell carcinoma; **(E)** Kidney renal clear cell carcinoma; **(F)** Kidney renal papillary cell carcinoma; **(G)** Liver hepatocellular carcinoma; **(H)** Lung adenocarcinoma; **(I)** Prostate adenocarcinoma; **(J)** Thyroid carcinoma; **(K)** Uterine Corpus Endometrial Carcinoma.

A locus-by-locus differential DNA methylation analysis was performed to define differentially methylated CpG sites between tumor tissues and matched adjacent normal tissues for each cancer type. The enzyme network from the KEGG database and the protein–protein interaction network from the STRING database were integrated using the Met-express algorithm to identify the hub genes within the regulatory network. For each cancer type, the significant differentially methylated CpG sites located in the hub genes have greatly changed, in which the epi-driver genes were defined as the genes with significant methylation (Supplementary Figure S1). The result shows that the numbers of significant CpG sites located in epi-driver genes ranged from 104 for THCA to 13,770 for UCEC, with other cancer types having between 302 and 6,810 CpG sites (Supplementary Table S2). The exploratory two-dimensional (2D) hierarchical clustering was analyzed based on these CpG sites. The DNA methylation profiles of tumors and matched adjacent normal tissues resulted in separate clusters, indicating a substantial difference in DNA methylation profiles between tumor, and non-tumor samples (Figure 2). For the differentially methylated CpG sites, we also investigated whether or not they corresponded to genes with associated CpG islands (including Island, N_Shore/Shelf, and S_Shore/Shelf). In the promoter regions (defined as the region 1.5 kb upstream or downstream of the nearest transcription start site), the significant differentially methylated CpG sites in all cancer types were located in CpG islands remarkably. On the contrary, the significant methylated CpG sites across various cancer types were located within the open-sea regions in the gene body (Fisher’s exact test, *p* < 2.2 × 10^−16^) (Supplementary Figure S2). Furthermore, the distribution of CpG sites showing different methylation was significantly enriched within high CpG island density regions, such as Island regions.

**FIGURE 3:**
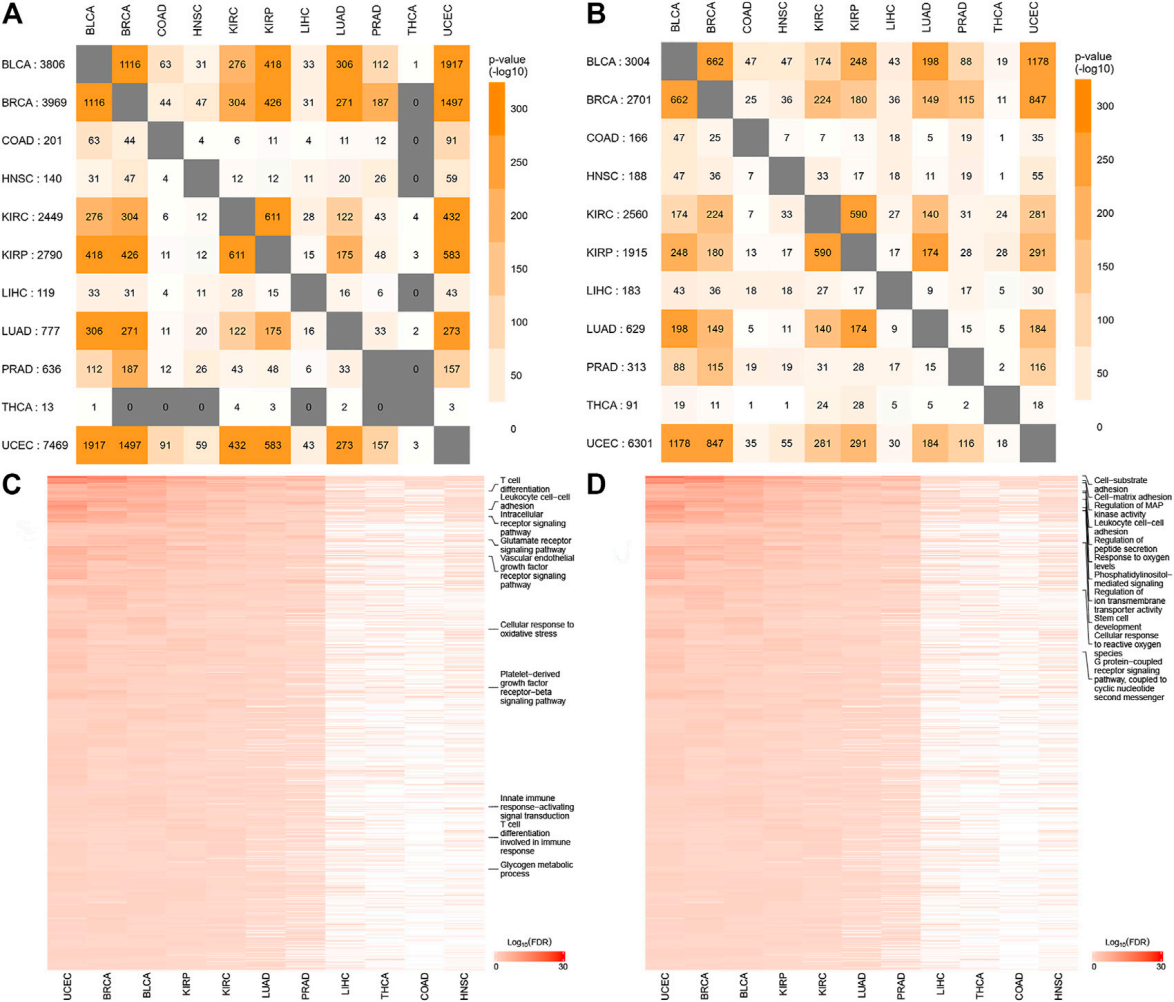
The significantly methylated CpG sites and Gene Ontology (GO) terms shared among the cancer type-specific DNA methylation signatures. For both the CpG sites hyper-methylated in tumor tissues for at least one cancer type **(A)** and the CpG sites hypo-methylated in tumor tissues for at least one cancer type **(B)**, the numbers of overlapping CpG sites between any two cancer types are shown, along with the significance of overlap (using colorgram, and by one-sided Fisher`s exact test); **(C)** GO terms significantly enriched for at least three cancer types (enrichment for cancer type defined as FDR <5% using one-sided Fisher`s exact test) within the respective sets of the key enzyme genes hyper-methylated in tumor tissues; **(D)** GO terms significantly enriched for at least three cancer types within the respective sets of the key-enzyme genes hypo-methylated in tumor tissues. Some GO terms significantly associated with tumorigenesis were enriched at least ten cancer types, such as T-cell differentiation, Glutamate receptor signaling pathway, Cellular response to reactive oxygen species, G protein-coupled receptor signaling pathway, and Glycogen metabolic process.

**FIGURE 4:**
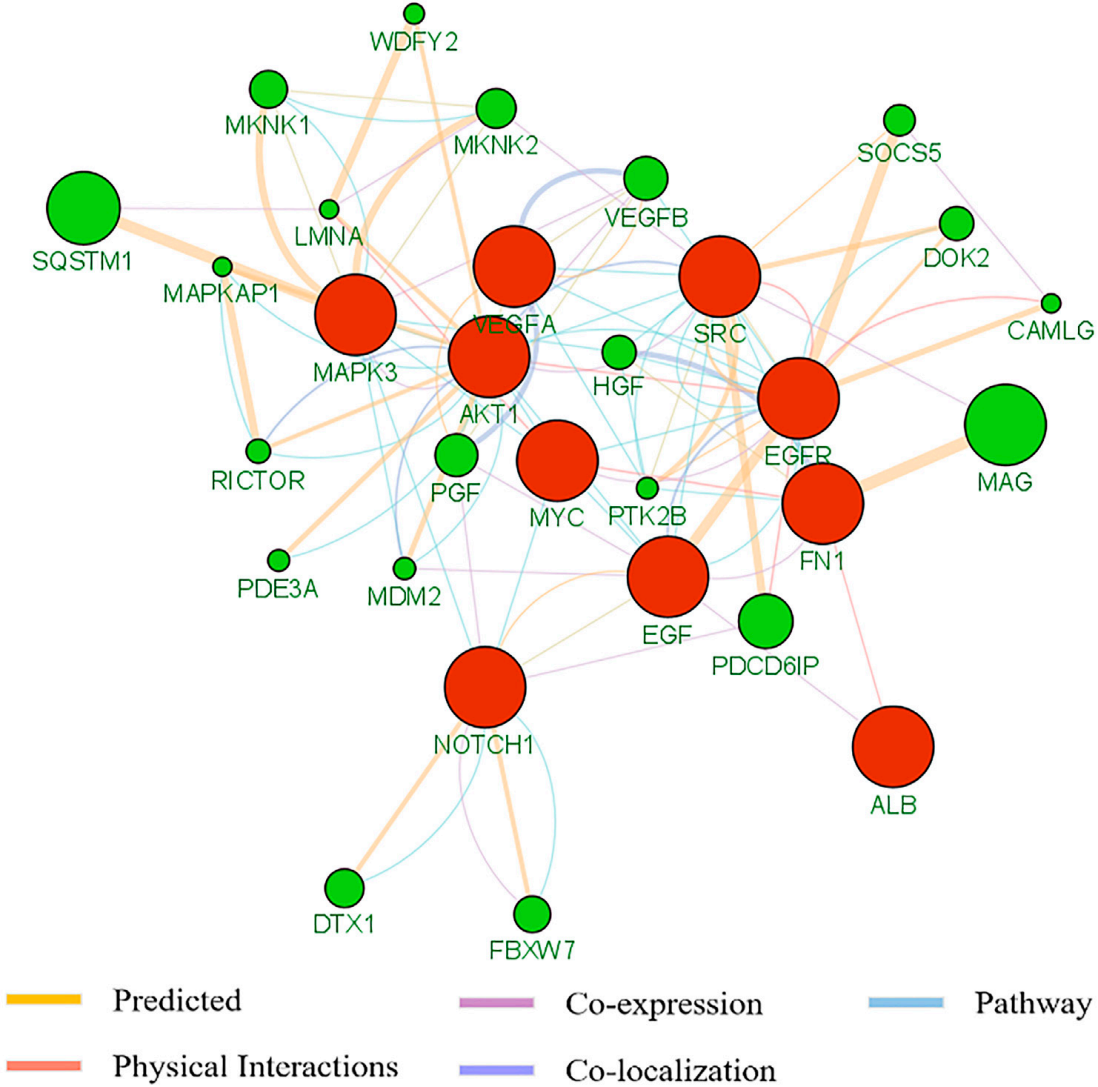
The protein–protein interaction of the top 10 genes based on degree within interaction network by means of the GeneMANA method. The weight of each edge was multiplied by the weight of the containing network. The size of the circle was defined as the score attribute, which indicated the relevance of each gene to the original list based on the selected networks. Higher scores suggested genes that were more likely to be functionally related. The shaded circles represented the key-enzyme genes significantly methylated.

To better understand the pan-cancer DNA methylation patterns, the abnormally methylated CpG sites for each cancer type were integrated to explore the methylation profiles. Although it was found that some differential methylation patterns related to cancer progression were shared among various cancer subtypes, each cancer subtype showed different cancer progression characteristics from other cancer subtypes. When comparing the methylation characteristics of cancer subtypes, some overlapping CpG sites were observed (Figures 3A,B). In many cases, the overlap of methylation characteristics between any two cancer subtypes was significant, even though the overlap itself involved a small number of CpG sites (e.g., on the order of 25%). In addition, we also found that a set of 730 CpG sites located within the epi-driver genes had the same direction of change for three or more cancer subtypes, including genes that had previously proved to have biological functions in cancer progression such as EGFR (Forloni et al., 2016; Apicella et al., 2018), NCOR2 (Bhasin et al., 2015), MAML3 (Sanchez-Vega et al., 2018), TSC2 (Papp et al., 2018), and FGFR2 (Roy et al., 2021) (Supplementary Table S3). We further examined whether any of the CpG sites located in epi-driver genes would be significantly enriched in tumor tissue-specific gene markers associated with the given cancer subtype [as obtained using The Network of Cancer Genes (NCG)] (Repana et al., 2019). Of 2,372 tissue-specific driver gene sets identified by *Repana*, only some statistically significant association (*p*-value < 0.001, one-side Hypergeometric test) was obtained. Function categories of CpG sites located in epi-driver genes were evaluated by using the Gene Ontology (GO) database (Supplementary Table S4). The remarkably enriched GO terms (FDR <5%, Fisher’s exact test) obtained with the cancer hyper-methylated genes for at least three cancer subtypes included “T cell differentiation”, “Leukocyte cell-cell adhesion”, “Intracellular receptor signaling pathway”, “Vascular endothelial growth factor receptor signaling pathway”, and “Glutamate receptor signaling pathway”. The significantly enriched GO terms based on the hypo-methylated genes for at least three cancer types included “Cell-substrate adhesion”, “Regulation of MAP kinase activity”, “G protein-coupled receptor signaling pathway, coupled to cyclic nucleotide second messenger”, “Stem cell development”, “Stem cell development”, and “Response to oxygen levels” (Figures 3C,D). We also integrated protein–protein interactions with these abnormal methylation genes based on the STRING database, which allowed us to explore the potential relationships. The top 10 genes based on degree within the interaction network involved EGFR, NOTCH1, VEGFA, ALB, SRC, AKT1, MYC, EGF, MAPK3, and FN1, which were in three or more cancer types. Based on the *GeneMANIA* method, the protein–protein interaction network was constructed, involving genes related to leukocyte migration, immune response-regulating cell surface receptor signaling pathway, and regulation of vascular endothelial growth factor receptor signaling pathway (Figure 4).

### Widespread Impact of DNA Methylation on Gene Expression Patterns

**FIGURE 5:**
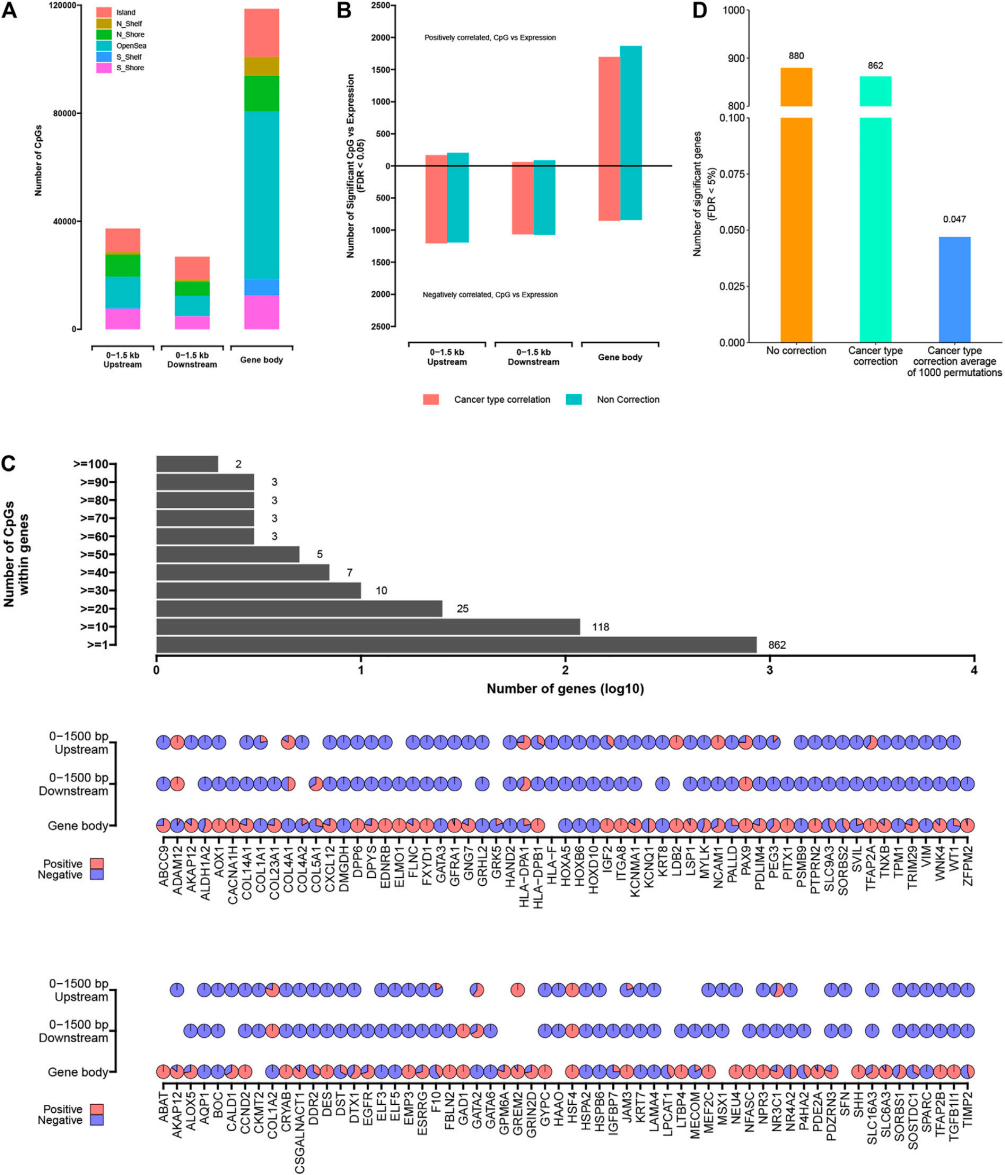
The significantly methylated CpG sites associated with altered expression of nearby genes. **(A)** Numbers of significantly methylated CpG sites identified as occurring within a gene body, 0–1.5 kb upstream of transcription start sites, 0–1.5 kb downstream of transcription start sites. **(B)** For each of the CpG site sets from part **(A)**, numbers of significant genes (FDR <5%), showing correlation between expression and associated methylation event. Numbers above and below zero point of *y*-axis denote positively and negatively correlated genes, respectively. Linear regression models also evaluated significant associations when correcting for cancer type (red). **(C)** Bar and pie plot of significance patterns for genes from part **(B)** (from the model correcting for cancer type). Bar plot shows a cumulative number of genes containing more than a specific number of CpG sites; pie plot shows significance patterns of correlation within the different regions of partial genes (red, positive correlation; blue, negative correlation). **(D)** Permutation testing (randomly shuffling the methylation event profiles and computing correlation with expression 1,000 times).

As may have been expected (Zhang et al., 2020; Zhang et al., 2018), the significantly methylated CpG sites could be related to gene expression patterns, while DNA methylation patterns associated with gene expression may be balanced or unbalanced within the up-/downstream of TSS sites or intergenic region. Therefore, a systematic pan-cancer analysis of all epi-driver genes was performed to explore gene expression patterns affected by abnormal DNA methylation. We aimed to identify some genes whose abnormal expression patterns were significantly related to abnormal DNA methylation near those genes (based on an analysis of 1,180 cases with both DNA methylation and RNA-seq data available). Because the DNA hyper-methylation sites in the nearby region of TSS sites were previously associated with its downregulation of gene expression in cancers (Gkountela et al., 2019; Zhao et al., 2020), we also explored the fixed windows at different positions away from CpG sites in the genome. Particularly, we have taken into consideration CpG sites occurring 0–1,500 bp upstream of TSS sites, 0–1,500 bp downstream of TSS sites, an intergenic region (IGR or Open-Sea), and within the gene body (Figure 5A). As compared to all CpG sites, CpG sites within 1,500 bp up-/downstream of TSS sites were significantly enriched for CpG islands involving Island, Shelf, or Shore, while CpG sites within the gene body were significantly enriched for the intergenic region (Open-Sea). For each of these regions, we evaluated the correlation between DNA methylation status and the expression patterns of genes associated with those CpG sites. Because each cancer subtype as a group would have a unique molecular characteristic (Hoadley et al., 2014), the cancer subtypes were regarded as a covariant factor and used in our analysis using linear models.

For each genomic region associated with the gene under consideration (i.e., genes with at least three samples related to CpG site in a given region), the widespread associations between DNA methylation event and gene expression were found (Figure 5B; Supplementary Table S5). For gene body, 0–1,500 bp upstream, and 0–1,500 bp downstream regions, the numbers of significantly associated genes (Storey & Tibshirani 2003) (correcting for cancer type) were 539, 518, and 512, respectively. Considering each of the above gene sets, there were more genes negatively correlated with DNA methylation events (i.e., when hyper-methylation events existed, the gene expression was downregulated) than positively correlated genes, except for the abnormal DNA methylation occurring within the gene body, in which more genes were positively correlated (1,697 CpG sites located within 333 genes) compared with negatively correlated genes (851 CpG sites located within 346 genes). On the other hand, we found more genes related to DNA methylation when cancer subtypes were not used for correction (Figure 5B), reflecting DNA methylation events as being strongly associated with cancer type. Based on the comprehensive analysis, we also found that the abnormal expression of a given gene was affected by multiple CpG sites with different degrees of methylation (Figure 5C), which revealed the complex regulation mechanism of DNA methylation.

In order to further prove the non-random correlation between DNA methylation events and gene expression, the permutation testing was carried out. For the entire window 0–1,500 up-/downstream of TSS sites to gene body associated with genes, the DNA methylation matrix was established by annotating the methylation level related to the given region for each sample. In each of the 1,000 tests, the DNA methylation profiles were randomly shuffled and the correlations associated with gene expression were computed. In the real dataset, 862 genes were found to have significant correlation (FDR < 0.05) after correcting for cancer subtypes. However, the permutation results obtained an average of 0.047 “significant” genes with an SD of 0.012 (Figure 5D). These results confirmed that although each of the two data platforms involved the biological and technical noise, a lot of the significant genes observed using the actual datasets would be unexplainable by multiple testing or noise.

### Key Epi-Driver Genes in Cancer Affected by DNA Methylation

The abnormally expressed genes related to nearby CpG sites included many previously reported cancer-related genes (Figure 5C). The downregulated genes associated with the abnormal methylation included GATA3 (participates in activating the Th1 and Th2 cell differentiation), IGF2 (within the PI3K–Akt signaling pathway), CACNA1H (within the MAPK signaling pathway), WT1 (within chaperones that modulate the interferon signaling pathway), and ADH1B (participate in Glycolysis/Gluconeogenesis). Those results showed that the abnormal DNA methylation events would presumably have a role in disrupting expression of the important epi-driver genes; for other genes, the abnormal DNA methylation events associated with genes could affect genomic regulatory elements. By analyzing the biological functions of 862 genes that were significantly related to abnormal DNA methylation located in 0–1,500 bp up-/downstream of TSS sites or within gene body (significant for any of the promoter regions in Figures 5A–C), gene functions by GO analysis included regulation of angiogenesis (71 genes), epithelial cell proliferation (71 genes), positive regulation of cell adhesion (63 genes), cell growth (50 genes), protein kinase B signaling (38 genes), and so on (Supplementary Figure S3; Supplementary Table S6). GO analysis showed that abnormal methylation events located within the epi-driver genes played an important role in the migration and deterioration of tumor cells. At the same time, DNA methylation, as a reversible event that did not change DNA sequence, provided the possibility for finding reliable therapeutic targets.

**FIGURE 6:**
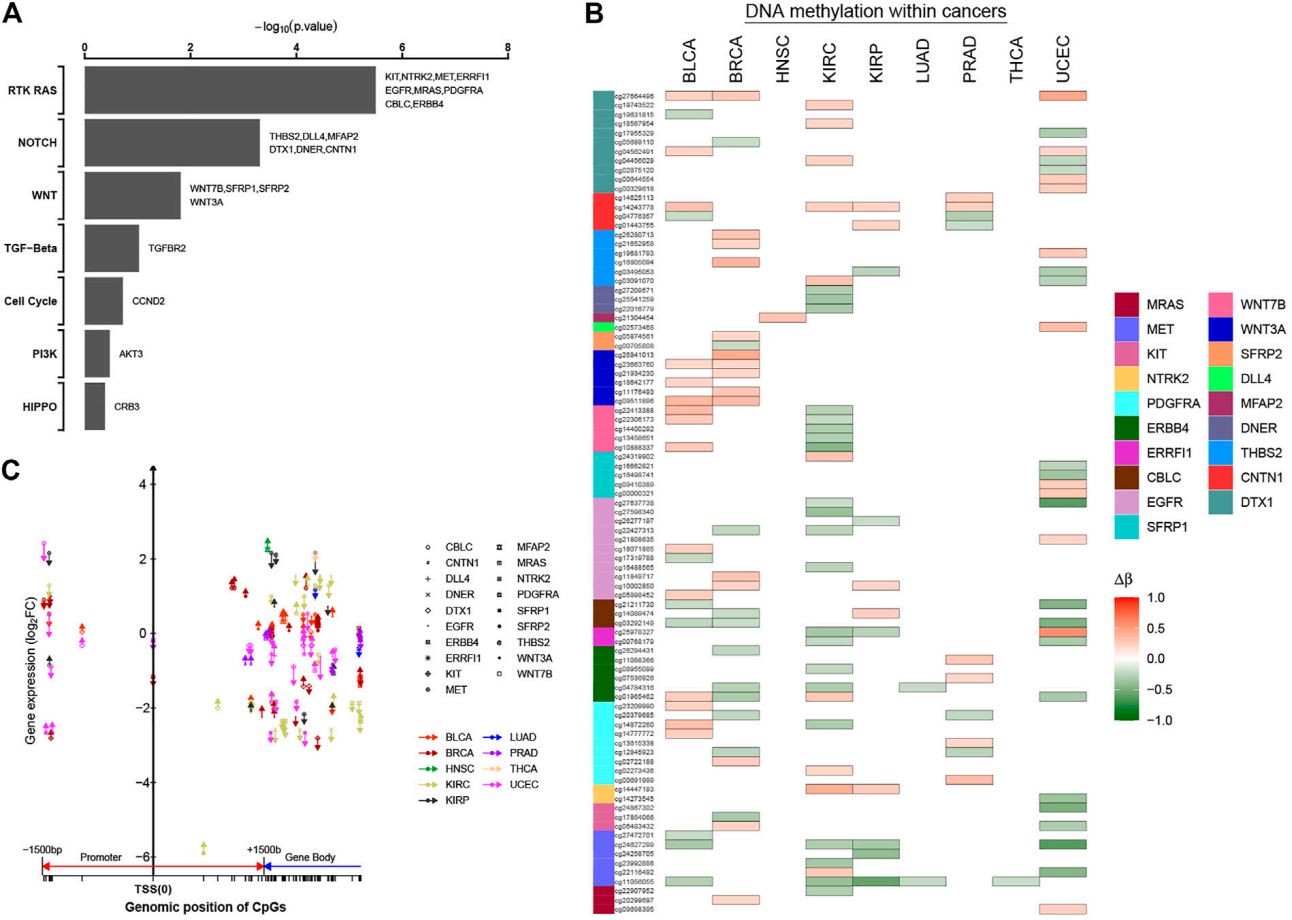
Significantly methylated CpG sites associated with key oncogenic or tumor-suppressive pathways. **(A)** For selected predefined pathways, significantly enriched oncogenic or tumor-suppressive pathways for genes significantly correlated with occurrence of 0–1.5 kb upstream of transcription start sites, 0–1.5 kb downstream of transcription start sites, and gene body. *p*-value by one-sided Fisher`s exact test. **(B, C)** Patterns of methylation event for selected genes from part **(A)**. Δβ was the differential methylation patterns (Δβ > 0, hyper-methylation in tumor tissues; Δβ < 0, hypo-methylation in the tumor tissues). In part **(C)**, the patterns of methylation event located within different regions for selected genes are shown (upward arrows, hyper-methylation in tumor tissues; downward arrows, hypo-methylation in tumor tissues).

As another approach to identify cancer-related genes affected by DNA methylation events, we focused on genes in the specific tumor-associated functional pathways. Previously, pathway-level alterations—according to somatic mutation, CAN, or epigenetic silencing—were investigated on the TCGA database, including chromatin modification, the mammalian target of rapamycin (mTOR), the SWI/SNF complex, receptor tyrosine kinase (RTK) signaling, p53-related (e.g., TP53, RB1), Wnt/*β*-catenin, and MYC (Chen et al., 2017; Zhang et al., 2017; Chen et al., 2018). We have identified numerous tumor-associated alterations in cancer epi-driver genes both at the level of DNA methylation and gene expression. Subsequently, we also asked whether there were significant changes in the tumor-associated patterns also in particular oncogenic signaling pathways. Using one-sided Fisher’s exact test, we identified seven out of 10 signaling pathways, highlighting differences in oncogenic pathways that may be imported in cancer initiation and progression (Figure 6A; Supplementary Table S7). Nineteen genes within three cancer-associated pathways showed statistical significance (*p*-value < 0.05), with the genes involving more CpG sites (Figure 6B). At the same time, we found that these DNA methylation sites not only were located in the promoter regions, but also exist in the gene body. Furthermore, most methylation sites were located in the promoter regions and gene body in the same genes, such as MET gene involving six abnormal methylation sites. For MET gene, one CpG site was located in the promoter region, and five CpG sites were located in the gene body. Those CpG sites with abnormal methylation in the same genes affected gene expression together (Figure 6C; Supplementary Table S8). Thus, results have shown that the regulatory mechanism of methylation has high complexity, and the abnormal methylation of the non-promoter region will also provide a novel stratification strategy and perspectives for cancers.

### Feature Selection and Tissue-specific DNA Methylation Biomarkers

**FIGURE 7:**
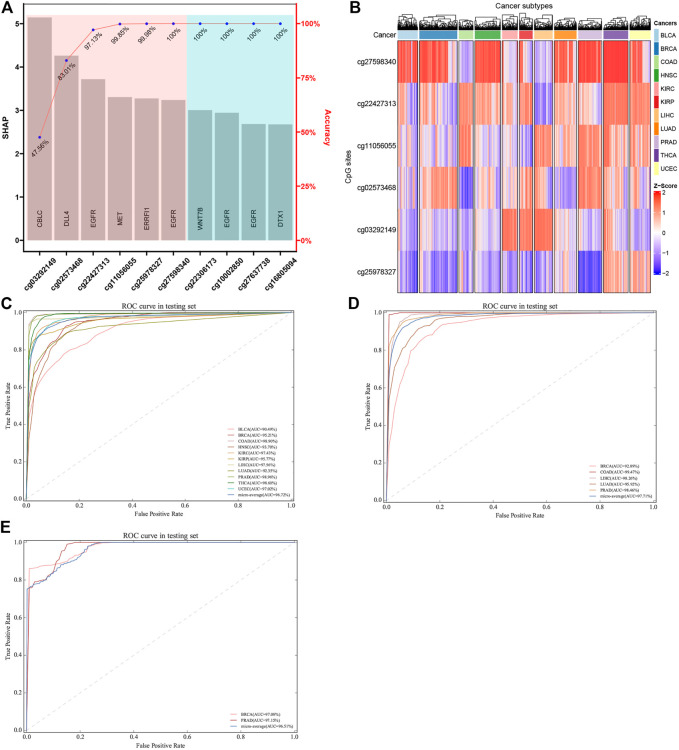
Construction and validation of tumor-specific classifier based on CpG sites. **(A)** Features were selected using SHAP and XGBoost algorithm. The features were ordered based on SHAP values, and the number of best features (biomarkers) was determined using accuracy based on XGBoost algorithm. **(B)** The hierarchical clustering was executed by means of Euclidean distance based on the biomarkers. *Z*-score was calculated based on the methylation levels of the biomarkers. **(C, D)** ROC of tumor-specific classifier with methylation biomarkers in the TCGA datasets and GEO datasets, respectively. **(E)** Two independent datasets with metastatic cancers were used, which involved GSE58999, GSE73549, and GSE38240. The original sites of these samples were breast and prostate tissues, which are located in lymph node tissue.

In order to obtain a classifier with better performance at distinguishing the primary sites of cancer, it is crucial to screen out the true tissue-specific features. Therefore, various strategies were used to filter out the highly tissue-specific biomarkers from omics datasets. Another factor to be considered in feature selection was the feature size of different omics datasets. For DNAm-based datasets, we used the primary feature selection to ensure that the optimal tissue-specific CpG sites (52 CpG sites within 23 genes) were identified, and these CpG sites showed an important influence on the occurrence or deterioration of tumors (Supplementary Table S7, S8). However, because the number of features obtained based on primary feature selection was still large, the secondary feature selection strategies (XGBoost and SHAP) were used to exclude redundant CpG sites. Based on the XGBoost and SHAP algorithms, we ranked each candidate biomarker. The higher the weight, the greater the contribution to the performance of classifier. Subsequently, each candidate biomarker was added to build classifier in turn, and the accuracy was calculated. When the top six CpG sites were combined to construct the classifier, the performance of the classifier reached a plateau. Finally, the optimal number of features (six CpG sites) was obtained using the automatic searching model of XGBoost and SHAP algorithms, because more features would lead to more complex model, but the performance of the classifier would not improve correspondingly, and even lead to over-fitting (Figure 7A). In order to verify the rationality of our feature selection method, we constructed a heatmap for the top six CpG sites of 11 tumor tissues of selected tissue-specific CpG sites for the DNAm-based profiles (Figure 7B). The figure showed that some tissues could be easily distinguished from other tissues, reflecting strong tissue specificity.

### Classifier Performance Evaluation

Actually, the performance of the classifier depended on the quality and quantity of the selected features. In this study, we used the balanced datasets and random forest algorithm to train classifier based on the best features (six CpG sites). In order to evaluate the performance of the classifier comprehensively, the random forest algorithm was compared with the other two benchmark classifiers (SVM and logistic regression algorithms). After optimizing the super parameters of each algorithm, we only recorded the best prediction results of three classifiers (random forest, SVM, and logistic regression classifiers). Apart from AUC, we also calculated the accuracy to provide more useful information about cancer subtype diagnosis (Table 2 **)**. The results showed that the random forest classifier had the best performance compared with the other two classifiers (SVM and logistic regression classifiers). Therefore, the subsequent analysis was based on the random forest classifier. We found that almost all the original tissues or cancer subtypes were correctly identified by the random forest classifier, and the overall testing accuracy reached 96.23% (CI 94.29%–98.17%) (Table 2; Figure 7C).

**TABLE 2:** Comparison of tissue-specific classifier based on the different algorithm.

Primary site	Testing dataset					
	SVM		RF		LR	
	ACC (%; 95% CI)	AUC (%; 95% CI)	ACC (%; 95% CI)	AUC (%; 95% CI)	ACC (%; 95% CI)	AUC (%; 95% CI)
BLCA	94.38 (93.71–95.06)	88.61 (85.79–91.44)	93.76 (93.00–94.51)	90.66 (88.45–92.86)	92.86 (91.83–93.89)	85.90 (84.49–87.31)
BRCA	94.34 (93.58–95.10)	93.40 (91.96–94.85)	94.46 (93.96–94.96)	95.33 (94.26–96.39)	91.73 (91.04–92.41)	87.02 (84.70–89.35)
COAD	99.10 (98.53–99.68)	99.52 (99.03–100.01)	98.75 (98.23–99.27)	99.34 (98.66–100.02)	98.05 (97.34–98.75)	99.44 (99.10–99.79)
HNSC	92.71 (91.88–93.53)	94.69 (93.81–95.57)	93.13 (92.03–94.23)	93.74 (92.28–95.20)	90.99 (90.42–91.55)	87.42 (86.13–88.72)
KIRC	96.84 (96.27–97.41)	96.77 (95.34–98.19)	96.33 (95.52–97.14)	97.55 (96.47–98.63)	91.81 (91.25–92.37)	92.77 (91.25–94.28)
KIRP	96.76 (96.15–97.38)	94.87 (93.71–96.02)	97.39 (96.94–97.83)	96.09 (94.56–97.62)	95.98 (95.38–96.58)	96.66 (95.22–98.09)
LIHC	99.06 (98.52–99.60)	98.17 (96.19–100.15)	98.44 (97.98–98.90)	97.96 (96.02–99.89)	99.06 (98.63–99.49)	99.13 (97.90–100.36)
LUAD	94.97 (94.54–95.40)	94.94 (93.27–96.61)	94.58 (93.79–95.37)	92.51 (90.36–94.66)	90.87 (90.11–91.63)	88.00 (85.83–90.17)
PRAD	98.52 (97.96–99.08)	99.74 (99.56–99.92)	98.21 (97.35–99.06)	99.33 (98.62–100.03)	97.50 (96.54–98.46)	99.11 (98.62–99.61)
THCA	96.80 (96.17–97.43)	98.04 (96.77–99.32)	97.11 (96.59–97.64)	98.79 (98.40–99.18)	96.25 (95.56–96.94)	98.64 (98.20–99.08)
UCEC	96.22 (95.37–97.06)	96.90 (95.39–98.41)	96.29 (95.50–97.09)	97.23 (95.30–99.16)	93.95 (93.03–94.88)	94.58 (92.19–96.98)
Average	96.34 (94.94–97.73)	95.57 (93.82–98.12)	96.22 (94.90–97.55)	96.23 (94.29–98.17)	94.46 (92.23–96.49)	93.52 (89.81–97.22)

Note: SVM, support vector machines; RF, random forest algorithm; LR, logistic regression algorithm.

As far as biological bias was concerned, the primary feature selection was mainly based on the integrated analysis of multi-omics datasets, which selected some potential biomarkers that have an important impact on tumor progression. However, the secondary feature selection based on XGBoost and SHAP algorithms was explained mathematically. In the actual scene, the datasets for detailed analysis are divided into two types: the first is that the dimension (D) of the data is smaller than the sample size (*n*); the second is that the dimension (D) of the data may be the same as or larger than the sample size (*n*). With the rapid development of biotechnology, biological data belonging to the second data model (D > n) have appeared and accumulated in large quantities (Negahban et al., 2010). Among them, epigenome data (such as DNA methylation data) are a typical biological data belonging to the second pattern. In order to use these data more effectively, two machine learning algorithms (XGBoost and SHAP) were used as a secondary feature selection to identify tissue-specific CpG sites from the primary feature selection (integration analysis of transcriptome, epigenome, and biological regulatory network). The random forest classifier constructed based on the secondary feature selection was shown to have much higher accuracy within most tissues, including those tissues that were difficult to be characterized by miRNA expression profiles. For example, Rolf et al. (Sokilde et al., 2014) constructed a tissue-specific classifier based on LASSO algorithm, which has relatively lower overall prediction accuracy (88% accuracy; CI 75%–94%) on 15 tissues. Especially for the colorectal tissue, the overall accuracy of LASSO classifier was 76.47%, while our six-CpG-based classifier had a higher performance on distinguishing cancer subtypes (99.29%) (Table 2). Similarly, the accuracy of our six-CpG-based classifier on distinguishing bladder tissue was 95.83%, while Rosenfeld et al.(2008) reported that the sensitivity of a miRNA-based classifier using K nearest-neighbor algorithm was zero. However, the testing samples and training samples were collected from the same public database (TCGA), which might lead to the technical illusion of such high prediction accuracy. If we collected other independent samples from a completely different public database, we might get a lower prediction accuracy. In fact, this possibility has also been considered. Our classifier was evaluated on five independent datasets collected from the GEO database, which is completely different from the TCGA database, and the results showed that our algorithm still had a higher performance on these testing samples.

We used five independent datasets coming from GEO to test the repeatability or generalization of the classifier, and the performances of our classifier based on GEO datasets are also shown in Table 3. The performance of our six-CpG-based classifier showed that those samples were correctly identified with an AUC of more than 95% (except for BRCA: 92.89%) (Figure 7D **)**. This result had further demonstrated that our integration method and algorithm had a very efficient prediction.

**TABLE 3:** Evaluation of tissue-specific classifier based on the independent datasets.

Tissues	Testing sets—01			Testing sets—02		
	GEO datasets	ACC (%; 95% CI)	AUC (%; 95% CI)	GEO datasets	ACC (%; 95% CI)	AUC (%; 95% CI)
BRCA	GSE69914	87.80 (86.74–88.87)	92.95 (91.59–94.31)	GSE58999	91.85 (90.70–93.01)	97.08 (96.72–97.45)
COAD	GSE48684	99.15 (98.34–99.95)	99.95 (99.89–100.00)			
LIHC	GSE89582	95.28 (94.29–96.27)	98.40 (97.48–99.32)			
LUAD	GSE66836	90.75 (89.70–91.80)	96.04 (95.01–97.07)			
PRAD	GSE73549	95.47 (94.63–96.32)	98.73 (98.07–99.40)	GSE38240	91.39 (89.84–92.93)	97.02 (96.70–97.34)

Note: Testing dataset 1 from the GEO database was used to evaluate the performance of tissue-specific classifier; testing dataset 2 from the GEO database was applied to evaluate the performance distinguishing the primary sites of metastatic cancers.

### Tumor Origin Detection on Metastatic Cancer Based on Multi-Omics

Although most cancer patients are primary tumors, 10%–15% of all cancers are diagnosed as metastatic tumors. Even after a comprehensive radiation tumor physics (B-ultrasound, CT, MRI, and x-ray) examination, blood test, and histological evaluation, only one-third of the tumor tissue might be diagnosed as having a primary site (Greco 2013). Therefore, metastatic cancers with CUP account for 30%–60% of all cancer diagnoses, and were the seventh most common type of cancer, ranking lower than lung cancer, prostate cancer, breast cancer, cervical cancer, colon cancer, and stomach cancer. Since effective metastatic cancer treatment relies on the early identification of primary sites, patients with CUP have a poor prognosis, with a median survival time of 3–6 months and a 1-year survival rate of less than 25% (Geirsson et al., 2017). In addition, many patients with CUP are diagnosed as poorly differentiated adenocarcinoma, which makes morphological and immunohistochemical interpretation difficult (Sokilde et al., 2014). Therefore, the identification of primary sites is crucial for choosing the best treatment strategy for patients with CUP. In order to further confirm whether the cancer tissue-specific model constructed based on the integration strategy can be used to predict the primary site of metastatic cancer, we used some cancer samples that have already metastasized for testing. Due to the limited amount of methylation chip datasets with metastatic cancer, it was impossible to test all metastatic tumor subtypes. In our research, we used three independent GEO datasets, which involved GSE58999, GSE73549, and GSE38240. The original sites of these samples were breast and prostate tissues, which are located in lymph node tissue. The results showed that the AUC of the six-CpG-based classifier was greater than 97% (Table 3; Figure 7E **)**, while Ding et al.(2019) reported that the AUC of the 12-CpG-site-based classifier using logistic regression algorithm was from 70% to 90%. The results proved that the classifier constructed based on an integration strategy of multi-omics datasets can better identify tumor original sites. These findings provide greater potential for improving the diagnosis and treatment of patients with CUP other than the primary cancer.

## Discussion

The metastatic cancers of unknown primary (CUP) sites usually have relatively low survival rate and survival time, because the best treatment largely depends on the correct identification of the primary sites. Therefore, there have been many methods to improve the diagnostic pathology of CUP, including the immunohistochemical method originally used for tumor subtype analysis (Oien 2009), proteomic analysis (Bloom et al., 2007), reverse transcription PCR strategy (Horlings et al., 2008), and large-scale mRNA chip (Horlings et al., 2008). However, these methods have low sensitivity, have s complicated experimental operation, and have difficulty in identifying highly differentiated metastatic cancers (Ramaswamy et al., 2001; Rosenfeld et al., 2008; Sokilde et al., 2014; Tothill et al., 2005). DNA methylation is highly tissue-specific and can be used to classify tumor subtypes, such as adrenocortical carcinoma (Assie et al., 2014), acute myeloid leukemia (Cancer Genome Atlas Research et al., 2013), hepatocellular carcinoma (Xu et al., 2017), and other cancer of unknown primary sites (Moran et al., 2016). In addition, because the changes of epigenome (such as DNA methylation) are often reflected at the transcriptome level, integrating epigenome and transcriptome can broaden our understanding of the molecular mechanism of biomarkers. The integration of gene co-expression network and biological network (including the metabolic regulation network and protein–protein interaction network) further provides systematic insights into the changes in metabolic network strictly regulated by transcription network. These interconnected networks can potentially be used to identify the new therapeutic targets and biomarkers (Chakraborty et al., 2018). Based on the integration strategy of multi-omics data, we found that these significant differentially methylated CpG sites located within epi-driver genes have certain tissue specificity (Figures 3A,B). As we all know, DNA methylation on CpG islands located in the promoter region is related to gene silencing, but genome-wide research using large-scale parallel sequencing has been able to detect methylation sites other than CpG islands, thus extending the mechanism of epigenetics-mediated transcription regulation to interested genome regions. For example, Jones (2012) has shown that abnormal DNA methylation in the gene body was closely related to the occurrence of diseases and was often positively related to gene expression. Arechederra and other researchers found a large number of hyper-methylated and highly expressed genes located in CpG islands in the mouse hepatocyte model, and these significantly hyper-methylated CpG sites existed in 5′-UTR or gene body. It was worth noting that the epigenetic events of upregulation of gene expression caused by hyper-methylation also occurred in 56% of HCC patients, who belong to cell proliferation subtypes (Arechederra et al., 2018). In a study of prostate cancer, Zhao et al. found the significant association between abnormal DNA methylation located in intergenic regions and carcinogenesis based on an integrated study of whole-genome, whole-methylome, and whole-transcriptome sequencing (Zhao et al., 2020). In addition, abnormal DNA methylation located in the Shelf/Shore regions extending from CpG islands to 2 kb has been identified as the key element of gene regulation (Irizarry et al., 2009a), including enhancer region and alternative transcription sites (Heyn et al., 2016). In our study, some significant differentially methylated CpG sites also existed in gene body and intergenic regions, which led to the abnormal expression in the epi-driver genes (Figures 5, 6). Function enrichment analysis further showed that genes with abnormal methylation located in gene body or intergenic regions were also significantly associated with tumor progression (Supplementary Figure S3). Therefore, these epigenetic changes would be also the most attractive targets for cancer treatment or intervention.

Based on multi-omics data, we found some abnormal methylation sites (Supplementary Table S7, S8 **)** in epi-driver genes that are closely related to cancer progression. However, in order to build a model with strong generalization performance for identifying cancer subtypes, we need to further screen meaningful molecular features. After filtering by multi-omics strategy, the ensemble strategies (XGBoost and SHAP) were used to filter the important molecular markers. Ensemble strategies have the performance of complex machine learning algorithms and the intuitive understanding of simple classifiers, and they are powerful and easy to train (Sokilde et al., 2014). Subsequently, we obtained the six aberrant methylation sites located in the five epi-driver genes (Figure 7A). These genes are closely related to the occurrence and progression of cancer, such as EGFR and CBLC genes related to cancer cell proliferation and differentiation, and intercellular signal transduction and ubiquitination modification; DLL4 and ERRFI1 genes related to cell adhesion, cell migration, and angiogenesis; and MET gene associated with apoptosis and cell invasion. Then, based on the random forest algorithm, the average accuracy in the training datasets and testing datasets from TCGA were more than 96%. However, the accuracy of breast cancer in the independent datasets (GEO database) was 87.80%, which was lower than that of other malignant cancers. It might be caused by the differences in processing methods of different datasets and the morphological polymorphism, dedifferentiation, or poor differentiation of cancer tissues. In addition, the accuracy of colorectal cancer was 99.15% in independent datasets from the GEO database. However, Tang et al. constructed a random forest classifier based on miRNA features, while the accuracy of colorectal cancer was only 83.44% (Tang et al., 2018). Rolf et al. constructed a LASSO classifier based on miRNA features from 15 cancer subtypes, and its accuracy of colorectal cancer was lower (76.74%) (Sokilde et al., 2014). Finally, we also tested based on two independent metastatic datasets from GEO, and the AUC and accuracy of determining the primary sites of metastatic cancer were more than 97% and 90% (Figure 7E), respectively. To sum up, the multi-omics strategy can find the abnormal methylation sites located in the epi-driver genes and identify the primary sites of metastatic cancer with a higher accuracy or distinguish the subtypes of cancer tissues.

In summary, based on genome-wide methylation profile, we identified six CpG sites that can effectively identify the origin sites of metastatic cancers and distinguish the subtypes of cancer tissues with the help of multi-omics strategy, and constructed a diagnostic model with a better performance. Interestingly, six significantly abnormal methylation sites were located in different regions of the epi-driver genes, which indicated the complexity of epigenetic mechanism. Enrichment analysis showed that these genes were associated with the growth, proliferation, migration, and signal transduction of tumor cells. Furthermore, it was worth noting that the AUC of the six-CpG-based classifier was greater than 97% when identifying the primary sites of metastatic cancers. Altogether, our six-CpG-based model has shown good performance in both training and validation datasets and has shown great potential in the diagnosis of pan-cancer or metastatic cancers.

## Data Availability

The original contributions presented in the study are included in the article/Supplementary Material. Further inquiries can be directed to the corresponding author. Publicly available datasets were analyzed in this study, these can be found in The Cancer Genome Atlas (TCGA) and Gene Expression Omnibus database (GEO).

## References

[B1] Al-Shahib A. Breitling R. Gilbert D. (2005). Feature Selection and the Class Imbalance Problem in Predicting Protein Function from Sequence. Appl. Bioinformatics 4, 195–203. 10.2165/00822942-200504030-00004 16231961

[B2] Apicella M. Giannoni E. Fiore S. Ferrari K. J. Fernández-Pérez D. Isella C. (2018). Increased Lactate Secretion by Cancer Cells Sustains Non-cell-autonomous Adaptive Resistance to MET and EGFR Targeted Therapies. Cel Metab. 28, 848–865. 10.1016/j.cmet.2018.08.006 30174307

[B3] Arechederra M. Daian F. Yim A. Bazai S. K. Richelme S. Dono R. (2018). Hypermethylation of Gene Body CpG Islands Predicts High Dosage of Functional Oncogenes in Liver Cancer. Nat. Commun. 9, 3164. 10.1038/s41467-018-05550-5 30089774 PMC6082886

[B4] Assié G. Letouzé E. Fassnacht M. Jouinot A. Luscap W. Barreau O. (2014). Integrated Genomic Characterization of Adrenocortical Carcinoma. Nat. Genet. 46, 607–612. 10.1038/ng.2953 24747642

[B5] Bhasin J. M. Lee B. H. Matkin L. Taylor M. G. Hu B. Xu Y. (2015). Methylome-wide Sequencing Detects DNA Hypermethylation Distinguishing Indolent from Aggressive Prostate Cancer. Cel Rep. 13, 2135–2146. 10.1016/j.celrep.2015.10.078 PMC4684962 26628371

[B6] Bjerre M. T. Nørgaard M. Larsen O. H. Jensen S. Ø. Strand S. H. Østergren P. (2020). Epigenetic Analysis of Circulating Tumor DNA in Localized and Metastatic Prostate Cancer: Evaluation of Clinical Biomarker Potential. Cells 9, 1362. 10.3390/cells9061362 PMC7349912 32486483

[B7] Bloom G. C. Eschrich S. Zhou J. X. Coppola D. Yeatman T. J. (2007). Elucidation of a Protein Signature Discriminating Six Common Types of Adenocarcinoma. Int. J. Cancer 120, 769–775. 10.1002/ijc.22041 17131332

[B8] Breiman L. (2001). Random Forests. Machine Learn. 45, 5–32. 10.1023/A:1010933404324

[B9] Cai Q. Zhang P. He B. Zhao Z. Zhang Y. Peng X. (2020). Identification of Diagnostic DNA Methylation Biomarkers Specific for Early-Stage Lung Adenocarcinoma. Cancer Genet. 246-247, 1–11. 10.1016/j.cancergen.2020.08.002 32805686

[B10] Cancer Genome Atlas Research N. Ley T. J. Miller C. Ding L. Raphael B. J. Mungall A. J. (2013). Genomic and Epigenomic Landscapes of Adult De Novo Acute Myeloid Leukemia. N. Engl. J. Med. 368, 2059–2074. 10.1056/NEJMoa1301689 23634996 PMC3767041

[B11] Chakraborty S. Hosen M. I. Ahmed M. Shekhar H. U. (2018). Onco-Multi-OMICS Approach: A New Frontier in Cancer Research. Biomed. Res. Int. 2018, 1–14. 10.1155/2018/9836256 PMC6192166 30402498

[B12] Chen F. Zhang Y. Bossé D. Lalani A.-K. A. Hakimi A. A. Hsieh J. J. (2017). Pan-urologic Cancer Genomic Subtypes that Transcend Tissue of Origin. Nat. Commun. 8, 199. 10.1038/s41467-017-00289-x 28775315 PMC5543131

[B13] Chen F. Zhang Y. Gibbons D. L. Deneen B. Kwiatkowski D. J. Ittmann M. (2018). Pan-Cancer Molecular Classes Transcending Tumor Lineage across 32 Cancer Types, Multiple Data Platforms, and over 10,000 Cases. Clin. Cancer Res. 24, 2182–2193. 10.1158/1078-0432.CCR-17-3378 29440175 PMC5932236

[B14] Chen J. Ma M. Shen N. Xi J. J. Tian W. (2013). Integration of Cancer Gene Co-expression Network and Metabolic Network to Uncover Potential Cancer Drug Targets. J. Proteome Res. 12, 2354–2364. 10.1021/pr400162t 23590569

[B16] Díaz-Uriarte R. Alvarez de Andrés S. (2006). Gene Selection and Classification of Microarray Data Using Random forest. BMC Bioinformatics 7, 3. 10.1186/1471-2105-7-3 16398926 PMC1363357

[B17] Ding W. Chen G. Shi T. (2019). Integrative Analysis Identifies Potential DNA Methylation Biomarkers for Pan-Cancer Diagnosis and Prognosis. Epigenetics 14, 67–80. 10.1080/15592294.2019.1568178 30696380 PMC6380428

[B18] Forloni M. Gupta R. Nagarajan A. Sun L.-S. Dong Y. Pirazzoli V. (2016). Oncogenic EGFR Represses the TET1 DNA Demethylase to Induce Silencing of Tumor Suppressors in Cancer Cells. Cel Rep. 16, 457–471. 10.1016/j.celrep.2016.05.087 PMC4945411 27346347

[B19] Geirsson Á. Valtysdottir S. T. Valtýsdóttir S. Sigvaldason A. Sigurðardóttir M. (2017). Æxli Af Óþekktum Toga: Tilfelli. Lbl 2017, 185–187. 10.17992/lbl.2017.04.132

[B20] Gkountela S. Castro-Giner F. Szczerba B. M. Vetter M. Landin J. Scherrer R. (2019). Circulating Tumor Cell Clustering Shapes DNA Methylation to Enable Metastasis Seeding. Cell 176, 98–112. 10.1016/j.cell.2018.11.046 30633912 PMC6363966

[B21] Greco F. A. (2013). Cancer of Unknown Primary Site: Improved Patient Management with Molecular and Immunohistochemical Diagnosis. Am. Soc. Clin. Oncol. Educ. Book 2013, 175–181. 10.14694/EdBook_AM.2013.33.175 23714493

[B22] Hao X. Luo H. Krawczyk M. Wei W. Wang W. Wang J. (2017). DNA Methylation Markers for Diagnosis and Prognosis of Common Cancers. Proc. Natl. Acad. Sci. USA 114, 7414–7419. 10.1073/pnas.1703577114 28652331 PMC5514741

[B23] Heyn H. Vidal E. Ferreira H. J. Vizoso M. Sayols S. Gomez A. (2016). Epigenomic Analysis Detects Aberrant Super-enhancer DNA Methylation in Human Cancer. Genome Biol. 17, 11. 10.1186/s13059-016-0879-2 26813288 PMC4728783

[B24] Hoadley K. A. Yau C. Wolf D. M. Cherniack A. D. Tamborero D. Ng S. (2014). Multiplatform Analysis of 12 Cancer Types Reveals Molecular Classification within and across Tissues of Origin. Cell 158, 929–944. 10.1016/j.cell.2014.06.049 25109877 PMC4152462

[B25] Horlings H. M. van Laar R. K. Kerst J.-M. Helgason H. H. Wesseling J. van der Hoeven J. J. M. (2008). Gene Expression Profiling to Identify the Histogenetic Origin of Metastatic Adenocarcinomas of Unknown Primary. J. Clin. Oncol. 26, 4435–4441. 10.1200/JCO.2007.14.6969 18802156

[B26] Irizarry R. A. Ladd-Acosta C. Wen B. Wu Z. Montano C. Onyango P. (2009a). The Human colon Cancer Methylome Shows Similar Hypo- and Hypermethylation at Conserved Tissue-specific CpG Island Shores. Nat. Genet. 41, 178–186. 10.1038/ng.298 19151715 PMC2729128

[B27] Irizarry R. A. Ladd-Acosta C. Wen B. Wu Z. Montano C. Onyango P. (2009b). The Human colon Cancer Methylome Shows Similar Hypo- and Hypermethylation at Conserved Tissue-specific CpG Island Shores. Nat. Genet. 41, 178–186. 10.1038/ng.298 19151715 PMC2729128

[B28] Jjingo D. Conley A. B. Yi S. V. Lunyak V. V. Jordan I. K. (2012). On the Presence and Role of Human Gene-Body DNA Methylation. Oncotarget 3, 462–474. 10.18632/oncotarget.497 22577155 PMC3380580

[B29] Jones P. A. (2012). Functions of DNA Methylation: Islands, Start Sites, Gene Bodies and beyond. Nat. Rev. Genet. 13, 484–492. 10.1038/nrg3230 22641018

[B30] Kirby M. K. Ramaker R. C. Roberts B. S. Lasseigne B. N. Gunther D. S. Burwell T. C. (2017). Genome-wide DNA Methylation Measurements in Prostate Tissues Uncovers Novel Prostate Cancer Diagnostic Biomarkers and Transcription Factor Binding Patterns. BMC Cancer 17, 273. 10.1186/s12885-017-3252-2 28412973 PMC5392915

[B31] Langfelder P. Horvath S. (2008). WGCNA: an R Package for Weighted Correlation Network Analysis. BMC Bioinformatics 9, 559. 10.1186/1471-2105-9-559 19114008 PMC2631488

[B32] Liu M. Shi T. (2017). Ten Years of Achievements in Biological and Medical Sciences. Sci. China Life Sci. 60, 111–115. 10.1007/s11427-017-9003-3 28215028

[B33] Massard C. Loriot Y. Fizazi K. (2011). Carcinomas of an Unknown Primary Origin-Diagnosis and Treatment. Nat. Rev. Clin. Oncol. 8, 701–710. 10.1038/nrclinonc.2011.158 22048624

[B34] Moran S. Martínez-Cardús A. Sayols S. Musulén E. Balañá C. Estival-Gonzalez A. (2016). Epigenetic Profiling to Classify Cancer of Unknown Primary: a Multicentre, Retrospective Analysis. Lancet Oncol. 17, 1386–1395. 10.1016/S1470-2045(16)30297-2 27575023

[B35] Negahban S. N. Ravikumar P. Wainwright M. J. Yu B. (2012). A Unified Framework for High-Dimensional Analysis of $M$-Estimators with Decomposable Regularizers. Statist. Sci. 27, 400. 10.1214/12-STS400

[B36] Noushmehr H. Weisenberger D. J. Diefes K. Phillips H. S. Pujara K. Berman B. P. (2010). Identification of a CpG Island Methylator Phenotype that Defines a Distinct Subgroup of Glioma. Cancer Cell 17, 510–522. 10.1016/j.ccr.2010.03.017 20399149 PMC2872684

[B37] Oien K. A. (2009). Pathologic Evaluation of Unknown Primary Cancer. Semin. Oncol. 36, 8–37. 10.1053/j.seminoncol.2008.10.009 19179185

[B38] Papp E. Hallberg D. Konecny G. E. Bruhm D. C. Adleff V. Noë M. (2018). Integrated Genomic, Epigenomic, and Expression Analyses of Ovarian Cancer Cell Lines. Cel Rep. 25, 2617–2633. 10.1016/j.celrep.2018.10.096 PMC6481945 30485824

[B39] Rajaraman P. Anderson B. O. Basu P. Belinson J. L. Cruz A. D. Dhillon P. K. (2015). Recommendations for Screening and Early Detection of Common Cancers in India. Lancet Oncol. 16, e352–e361. 10.1016/S1470-2045(15)00078-9 26149887

[B40] Ramaswamy S. Tamayo P. Rifkin R. Mukherjee S. Yeang C.-H. Angelo M. (2001). Multiclass Cancer Diagnosis Using Tumor Gene Expression Signatures. Proc. Natl. Acad. Sci. 98, 15149–15154. 10.1073/pnas.211566398 11742071 PMC64998

[B41] Rao X. Evans J. Chae H. Pilrose J. Kim S. Yan P. (2013). CpG Island Shore Methylation Regulates Caveolin-1 Expression in Breast Cancer. Oncogene 32, 4519–4528. 10.1038/onc.2012.474 23128390 PMC3787796

[B42] Repana D. Nulsen J. Dressler L. Bortolomeazzi M. Venkata S. K. Tourna A. (2019). The Network of Cancer Genes (NCG): a Comprehensive Catalogue of Known and Candidate Cancer Genes from Cancer Sequencing Screens. Genome Biol. 20, 1. 10.1186/s13059-018-1612-0 30606230 PMC6317252

[B43] Rosenfeld N. Aharonov R. Meiri E. Rosenwald S. Spector Y. Zepeniuk M. (2008). MicroRNAs Accurately Identify Cancer Tissue Origin. Nat. Biotechnol. 26, 462–469. 10.1038/nbt1392 18362881

[B44] Roy S. Singh A. P. Gupta D. (2021). Unsupervised Subtyping and Methylation Landscape of Pancreatic Ductal Adenocarcinoma. Heliyon 7, e06000. 10.1016/j.heliyon.2021.e06000 33521362 PMC7820567

[B45] Sanchez-Vega F. Mina M. Armenia J. Chatila W. K. Luna A. La K. C. (2018). Oncogenic Signaling Pathways in the Cancer Genome Atlas. Cell 173, 321–337. 10.1016/j.cell.2018.03.035 29625050 PMC6070353

[B46] Selamat S. A. Chung B. S. Girard L. Zhang W. Zhang Y. Campan M. (2012). Genome-scale Analysis of DNA Methylation in Lung Adenocarcinoma and Integration with mRNA Expression. Genome Res. 22, 1197–1211. 10.1101/gr.132662.111 22613842 PMC3396362

[B47] Søkilde R. Vincent M. Møller A. K. Hansen A. Høiby P. E. Blondal T. (2014). Efficient Identification of miRNAs for Classification of Tumor Origin. J. Mol. Diagn. 16, 106–115. 10.1016/j.jmoldx.2013.10.001 24211363

[B48] Statnikov A. Wang L. Aliferis C. F. (2008). A Comprehensive Comparison of Random Forests and Support Vector Machines for Microarray-Based Cancer Classification. BMC Bioinformatics 9, 319. 10.1186/1471-2105-9-319 18647401 PMC2492881

[B49] Storey J. D. Tibshirani R. (2003). Statistical Significance for Genomewide Studies. Proc. Natl. Acad. Sci. 100, 9440–9445. 10.1073/pnas.1530509100 12883005 PMC170937

[B50] Sui J. Wu X. Wang C. Wang G. Li C. Zhao J. (2021). Discovery and Validation of Methylation Signatures in Blood-Based Circulating Tumor Cell-free DNA in Early Detection of Colorectal Carcinoma: a Case-Control Study. Clin. Epigenet 13, 26. 10.1186/s13148-020-00985-4 PMC7856810 33536049

[B51] Tang W. Wan S. Yang Z. Teschendorff A. E. Zou Q. (2018). Tumor Origin Detection with Tissue-specific miRNA and DNA Methylation Markers. Bioinformatics 34, 398–406. 10.1093/bioinformatics/btx622 29028927

[B52] Tothill R. W. Kowalczyk A. Rischin D. Bousioutas A. Haviv I. van Laar R. K. (2005). An Expression-Based Site of Origin Diagnostic Method Designed for Clinical Application to Cancer of Unknown Origin. Cancer Res. 65, 4031–4040. 10.1158/0008-5472.CAN-04-3617 15899792

[B53] Vogelstein B. Papadopoulos N. Velculescu V. E. Zhou S. Diaz L. A. Jr. Kinzler K. W. (2013). Cancer Genome Landscapes. Science 339, 1546–1558. 10.1126/science.1235122 23539594 PMC3749880

[B54] Wang J. Han X. Sun Y. (2017). DNA Methylation Signatures in Circulating Cell-free DNA as Biomarkers for the Early Detection of Cancer. Sci. China Life Sci. 60, 356–362. 10.1007/s11427-016-0253-7 28063009

[B55] Wei J.-H. Haddad A. Wu K.-J. Zhao H.-W. Kapur P. Zhang Z.-L. (2015). A CpG-Methylation-Based Assay to Predict Survival in clear Cell Renal Cell Carcinoma. Nat. Commun. 6, 8699. 10.1038/ncomms9699 26515236 PMC4846314

[B56] Wu D. Gong C. Su C. (2017). Genome-wide Analysis of Differential DNA Methylation in Silver-Russell Syndrome. Sci. China Life Sci. 60, 692–699. 10.1007/s11427-017-9079-7 28624953

[B57] Wu L. Yang Y. Guo X. Shu X.-O. Cai Q. Shu X. (2020). An Integrative Multi-Omics Analysis to Identify Candidate DNA Methylation Biomarkers Related to Prostate Cancer Risk. Nat. Commun. 11, 3905. 10.1038/s41467-020-17673-9 32764609 PMC7413371

[B58] Xu R.-h. Wei W. Krawczyk M. Wang W. Luo H. Flagg K. (2017). Circulating Tumour DNA Methylation Markers for Diagnosis and Prognosis of Hepatocellular Carcinoma. Nat. Mater 16, 1155–1161. 10.1038/nmat4997 29035356

[B59] Yang X. Gao L. Zhang S. (2017). Comparative Pan-Cancer DNA Methylation Analysis Reveals Cancer Common and Specific Patterns. Brief Bioinform 18, bbw063–773. 10.1093/bib/bbw063 27436122

[B60] Zhang Y. Chen F. Chen F. Fonseca N. A. He Y. Fujita M. (2020). High-coverage Whole-Genome Analysis of 1220 Cancers Reveals Hundreds of Genes Deregulated by Rearrangement-Mediated Cis-Regulatory Alterations. Nat. Commun. 11, 736. 10.1038/s41467-019-13885-w 32024823 PMC7002524

[B61] Zhang Y. Kwok-Shing Ng P. Kucherlapati M. Chen F. Liu Y. Tsang Y. H. (2017). A Pan-Cancer Proteogenomic Atlas of PI3K/AKT/mTOR Pathway Alterations. Cancer Cell 31, 820–832. 10.1016/j.ccell.2017.04.013 28528867 PMC5502825

[B62] Zhang Y. Yang L. Kucherlapati M. Chen F. Hadjipanayis A. Pantazi A. (2018). A Pan-Cancer Compendium of Genes Deregulated by Somatic Genomic Rearrangement across More Than 1,400 Cases. Cel Rep. 24, 515–527. 10.1016/j.celrep.2018.06.025 PMC6092947 29996110

[B63] Zhao S. G. Chen W. S. Li H. Foye A. Zhang M. Sjöström M. (2020). The DNA Methylation Landscape of Advanced Prostate Cancer. Nat. Genet. 52, 778–789. 10.1038/s41588-020-0648-8 32661416 PMC7454228

