# Supplementary figures and images for: Pan-Cancer DNA Methylation Analysis and Tumor Origin Identification of Carcinoma of Unknown Primary Site Based on Multi-Omics
Source: Front Genet. 2022 Jan 6;12:798748. doi: 10.3389/fgene.2021.798748 (PMC8770539; doi:10.3389/fgene.2021.798748)

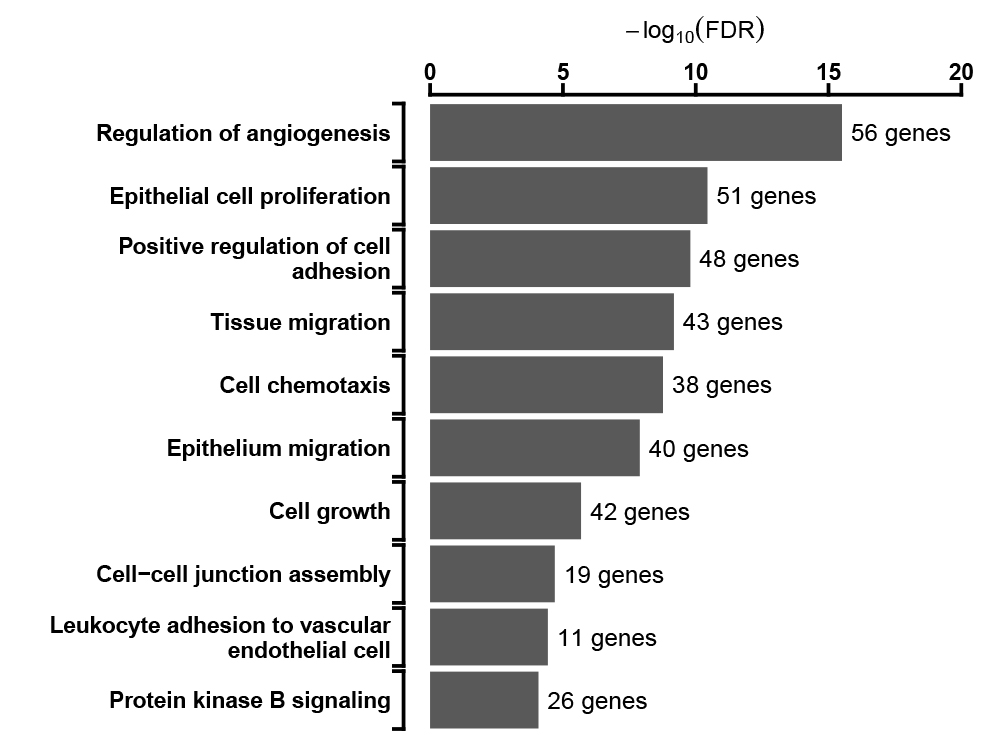

Supplement: Supplementary file 2 [file Image3.JPEG]

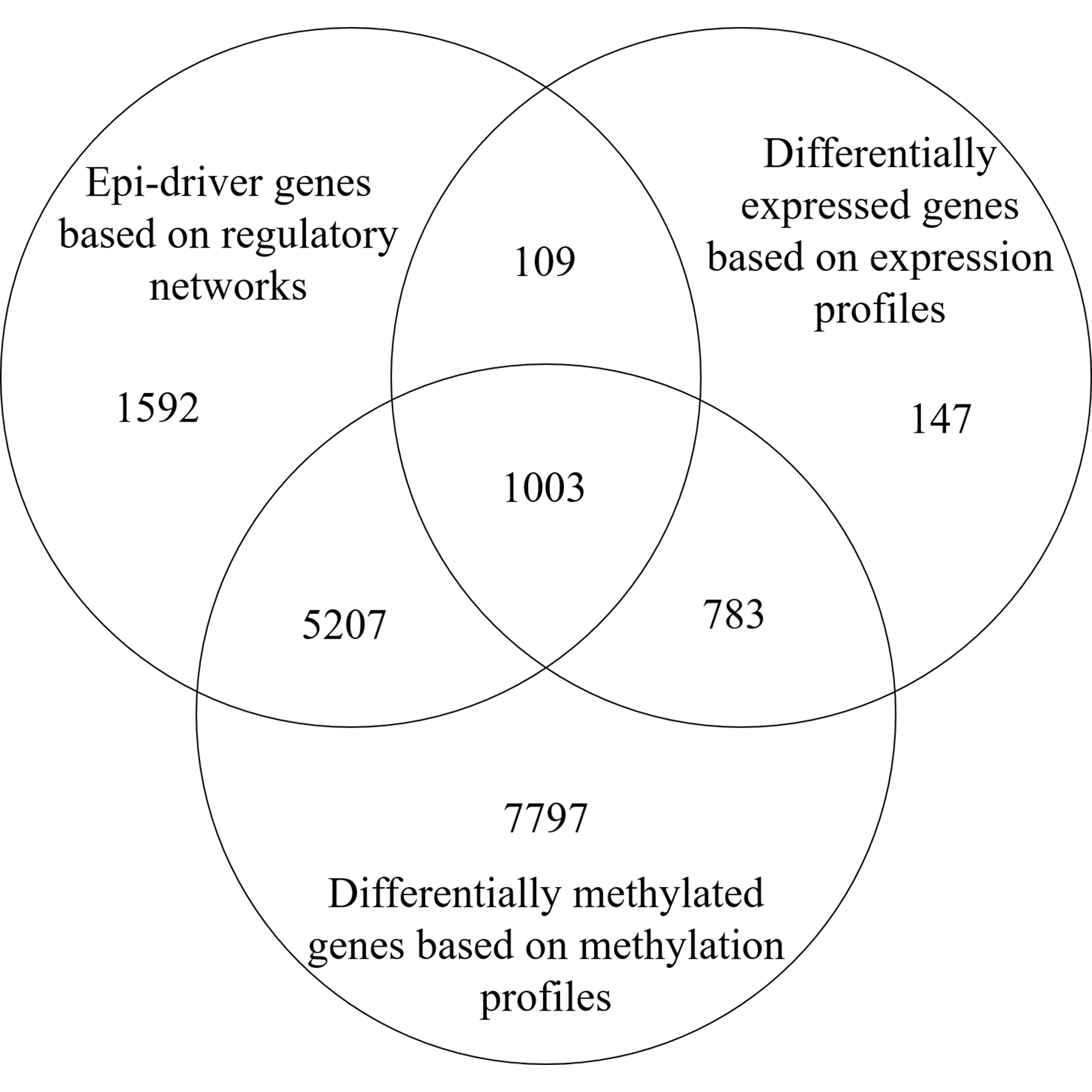

Supplement: Supplementary file 5 [file Image1.JPEG]

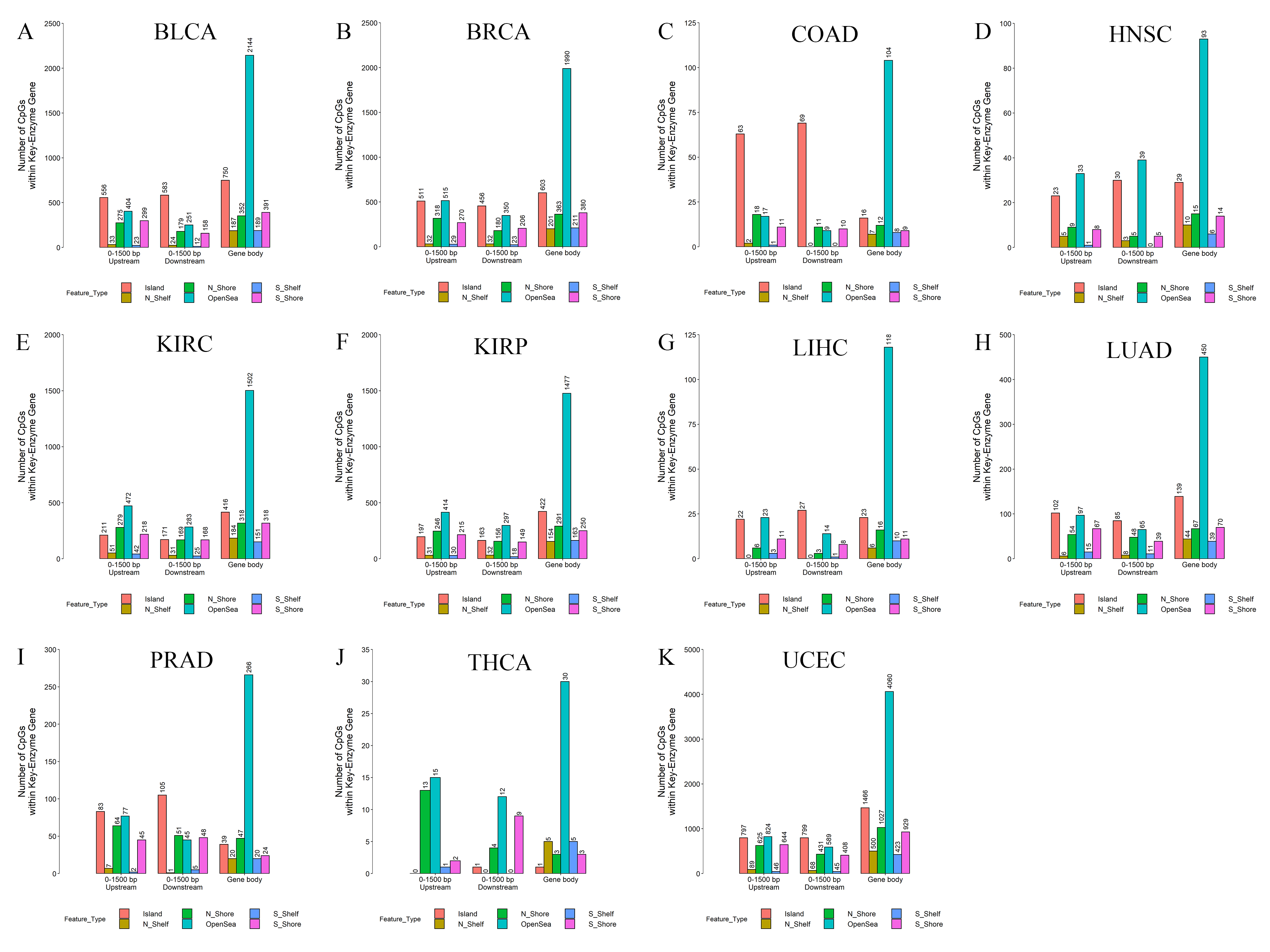

Supplement: Supplementary file 6 [file Image2.JPEG]
